# Application of Numerical Modeling and Finite Element Analysis in Fused Filament Fabrication: A Review

**DOI:** 10.3390/ma17174185

**Published:** 2024-08-23

**Authors:** Saeed Behseresht, Young Ho Park, Allen Love, Omar Alejandro Valdez Pastrana

**Affiliations:** Department of Mechanical and Aerospace Engineering, New Mexico State University, Las Cruces, NM 88003, USA; behsaeed@nmsu.edu (S.B.); alove@nmsu.edu (A.L.); pastrana@nmsu.edu (O.A.V.P.)

**Keywords:** additive manufacturing, fused deposition modeling, numerical modeling, finite element analysis

## Abstract

Additive manufacturing (AM) is not necessarily a new process but an advanced method for manufacturing complex three-dimensional (3D) parts. Among the several advantages of AM are the affordable cost, capability of building objects with complex structures for small-batch production, and raw material versatility. There are several sub-categories of AM, among which is fused filament fabrication (FFF), also commonly known as fused deposition modeling (FDM). FFF has been one of the most widely used additive manufacturing techniques due to its cost-efficiency, simplicity, and widespread availability. The FFF process is mainly used to create 3D parts made of thermoplastic polymers, and complex physical phenomena such as melt flow, heat transfer, solidification, crystallization, etc. are involved in the FFF process. Different techniques have been developed and employed to analyze these phenomena, including experimental, analytical, numerical, and finite element analysis (FEA). This study specifically aims to provide a comprehensive review of the developed numerical models and simulation tools used to analyze melt flow behavior, heat transfer, crystallization and solidification kinetics, structural analysis, and the material characterization of polymeric components in the FFF process. The strengths and weaknesses of these numerical models are discussed, simplifications and assumptions are highlighted, and an outlook on future work in the numerical modeling and FE simulation of FFF is provided.

## 1. Introduction

Additive manufacturing (AM) is a manufacturing technique that builds three-dimensional (3D) components in a layer-by-layer fashion, generally but not necessarily using 3D CAD modeling [1]. This technique blends materials through several mechanisms such as fusion, binding, or solidifying materials such as liquid resin, solid filaments, and powders. While AM has been utilized for processing materials for almost over two decades, it has only recently been recognized as an important commercial manufacturing technology [2]. Due to the inherent features of the layer-by-layer manufacturing procedure, AM is capable of minimizing raw material waste, with buy-to-fly ratios between 1:1 and 3:1 [3]. AM has the potential to exceptionally reduce the cost of manufacturing industrial components because it consumes far less feedstock material than traditional manufacturing techniques [3,4].

Fused filament fabrication (FFF), commonly known as fused deposition modeling (FDM) (both abbreviations will be used interchangeably throughout this paper), is an additive manufacturing technique used for manufacturing 3D components made of thermoplastic and thermoset polymers. In order to build a 3D component using this process, slicing software is used to slice the CAD file into layers for fabrication, control movement of the extruder head, extrusion, and other process parameters so that a complex part can be readily manufactured [5,6]. FFF has been frequently utilized in a variety of industries, including but not limited to aerospace, automotive, and construction [7,8]. Compared to conventional manufacturing techniques such as injection molding, FFF has many advantages. These advantages include, but are not limited to, the rapid manufacturing of small-scale components, the capability of manufacturing components with anisotropic properties, and the reduced cost of fixtures and molds. However, FFF-manufactured parts possess low mechanical properties compared to those produced by injection molding and other techniques, mainly due to insufficient polymer reptation for a fully entangled bulk micro-structure. Therefore, numerical models are essential to address this issue, enabling FFF to capture a portion of the small-batch production injection molding market by reducing tooling costs and lead times. During the FFF process, polymers, either pure or composite, are extruded from a nozzle in a molten state onto a build plate or on top of previously deposited layers and subsequently solidified to lower temperatures to produce the final solid components. This process encompasses heating and cooling, phase change, crystallization, and solidification [9]. The thermo-plasticity of the material plays an important role in FFF process, as it helps the molten material to fuse to previous layers of solidified material effectively [10,11]. Many parameters affect the quality and functionality of the final product; among them are print speed, extrusion temperature, layer height, and build plate temperature.

Controlling all print-related parameters, combined with a large number of thermal, physical, and mechanical properties of the material, in the FFF process makes trial and error a cost-inefficient procedure in manufacturing flawless components [12,13]. Numerical models and finite element analysis (FEA) have been effective methods to prevent such costs, as they can predict many unexpected errors by identifying sources of failure such as temperature gradients, residual stresses, warpage, and deformation in manufacturing processes, including FFF [14,15]. Even though numerical simulation methods have some drawbacks and difficulties in solving special problems such as non-uniform time scale problems, they remain highly cost-efficient and fast methods to study the physical phenomena involved in manufacturing processes compared to the time-consuming and costly “trial and error” experiments [16]. Due to advancements in numerical simulation technologies and computational capabilities, it is possible to predict and study the relationships among manufacturing process parameters, micro-structure evolution, and mechanical properties during additive manufacturing processes [17]. It is worth mentioning that numerical modeling and FEA are powerful tools for a wide range of analyses beyond AM [18,19,20].

**Figure 1:**
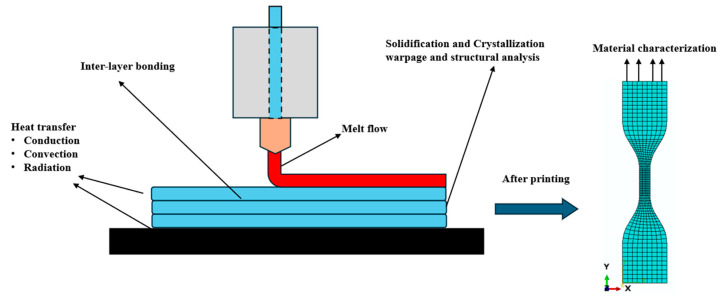
Schematic representation of the FFF process along with physical phenomena that can be numerically modeled.

As shown in Figure 1, during the FFF process, the raw filament is fed through a heated nozzle, undergoes a phase change, and melts. The behavior of this melt flow and the proposed numerical models are discussed in Section 2. The molten material is then deposited onto a print bed or previously deposited layers and starts to cool and solidify, which will be presented in detail in Section 3. Since the integrity and functionality of the final FFF-printed part depend directly on the efficient bonding between layers, bonding efficiency will be covered in Section 4. Throughout the FFF process, heat is dissipated to the surrounding environment through convection, radiation, etc., which makes thermal analysis a crucial aspect during the process. Thermal analysis will be covered in Section 5. During the FFF process, material shrinkage causes the printed components to deform and warp. Consequently, structural analysis is essential to predict possible failures while printing, and this will be presented in Section 6. Finally, material characterization, a key step in analyzing and comparing the mechanical properties of additively manufactured components, will be covered in Section 7.

Many researchers have proposed various numerical models and FE simulation techniques in their studies in order to address challenges in FFF additive manufacturing. As previously discussed, the FFF process involves several physical phenomena, including heat transfer, crystallization, solidification, deformation, etc. These phenomena can be simulated using FEA and numerical modeling. In this review paper, we aim to summarize available numerical and simulation-based models, their strengths and weaknesses, and their applications in the field of additive manufacturing. We reviewed more than 100 research papers and discussed available numerical and FE models representing different stages of the FFF process, including polymer melt flow behavior in the early stages of extrusion, heat transfer and thermal analysis, solidification and crystallization, inter-layer bonding, structural analysis, and the material characterization of printed components.

## 2. Melt Flow Behavior

**Figure 2:**
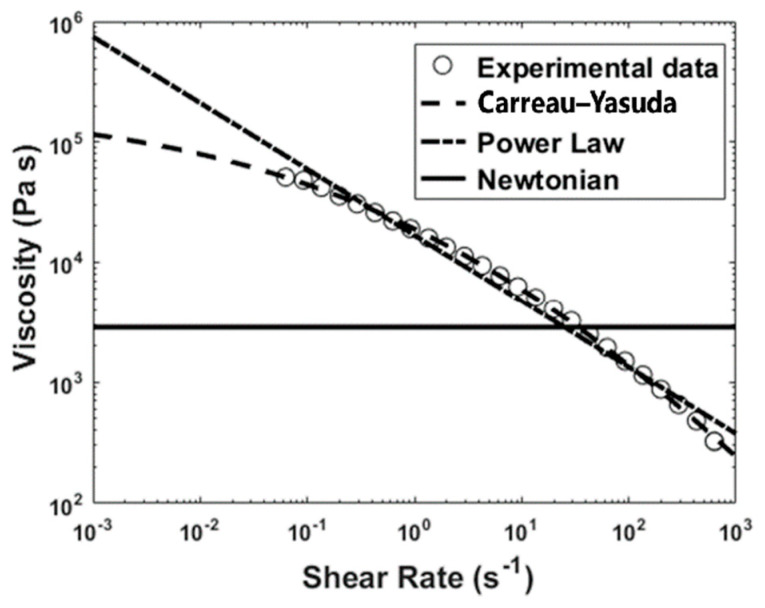
Viscosity as a function of shear rate for generalized Newtonian fluid models. Reprinted from Ref. [23].

The FFF process starts with the extrusion of polymers in a molten state through a cylindrical nozzle. Therefore, an important part of FFF process analysis is simulating the melt flow field in the extrusion nozzle of the printer [21]. Polymer melt flowing from extruder nozzle can generally be described by an isothermal, incompressible, non-Newtonian, creeping flow governed by the Navier–Stokes equations [22]. The conservation of mass and momentum under these assumptions is defined as:

(1) ∇⋅u=0

(2) $$\rho \frac{\partial u}{\partial t}=\nabla p+\nabla \cdot \tau$$

where *u* is the velocity of the polymer melt field, *τ* is the stress field inside the flow, *p* is the static pressure, *ρ* is the density of the material, and *t* is the time. There are different models to describe the rheological properties of molten polymers, such as viscosity. The viscosity of non-Newtonian fluids is generally considered a function of shear rate [23]. The shear-thinning behavior of a polymer melt can be described using the power law or the Carreau–Yasuda model, respectively, as follows:

(3) $$\eta (\dot{\gamma })=K{\left(\dot{\gamma }\right)}^{n-1}$$

(4) $$\eta (\dot{\gamma })=\eta_{\infty }+(\eta_{0}-\eta_{\infty }){\left(1+{\left(\kappa \dot{\gamma }\right)}^{a}\right)}^{\frac{n-1}{a}}$$

where *K* is the consistency index, *n* is the power law index, $\eta_{\infty }$ is the infinite-shear-rate viscosity, $\eta_{0}$ is the zero-shear-rate viscosity, and *k* is the natural time [23]. Different non-Newtonian and Newtonian models for the viscosity of ABS are shown in Figure 2.

The stress tensor *τ*, as the total internal stress of the polymer melt flowing through the extruder nozzle in Equation (2), can be decomposed into pure viscous stress ($\sigma_{s}$) and viscoelastic stress components ($\sigma_{v}$) utilizing the elastic viscous split stress method [24]:

(5) $$\tau =\sigma_{s}+\sigma_{v}$$

(6) $$\sigma_{s}=2\eta D$$

where *h* is viscosity and can be defined based on Equations (3) or (4), and *D* is the strain rate tensor, defined as

(7) $$D=\frac{\nabla v+{\left(\nabla v\right)}^{T}}{2}$$

In Equation (7), ∇v and ${\left(\nabla v\right)}^{T}$ are the velocity gradient and transpose of the velocity gradient, respectively.

There are several models to define the viscoelasticity of polymers in the molten state such as the Voigt model, Maxwell model, and Kelvin model. One of the frequently used expressions for the viscoelastic component of stress is the simplified viscoelastic model (SVM), which is defined as

(8) $$\sigma_{v}=\left[\begin{array}{ccc} \sigma_{xx} & \sigma_{xy} & 0\\ \sigma_{yx} & 0 & 0\\ 0 & 0 & 0 \end{array}\right]=\left[\begin{array}{ccc} \psi \mu (\dot{\chi })\dot{\chi } & \eta (\dot{\gamma })\dot{\gamma } & 0\\ \eta (\dot{\gamma })\dot{\gamma } & 0 & 0\\ 0 & 0 & 0 \end{array}\right]$$

The normal viscosity $\mu (\dot{\chi })$ can be defined in the same way as the shear viscosity $\eta (\dot{\gamma })$ by replacing $\dot{\chi }$ with $\dot{\gamma }$ in Equations (3) or (4) based on the model being used.

The simulation of non-Newtonian free-surface flows is an important topic of computational rheology since it has several applications in polymer processing techniques such as FFF [22]. Non-Newtonian fluids are usually characterized by two features: first, by a varying viscosity that depends on the flow conditions, as seen in shear-thinning fluids, and second, by a time-dependent stress response that includes an elastic stress component representing the recoverable deformations from the stretching of polymer chains, as in viscoelastic liquids.

**Figure 3:**
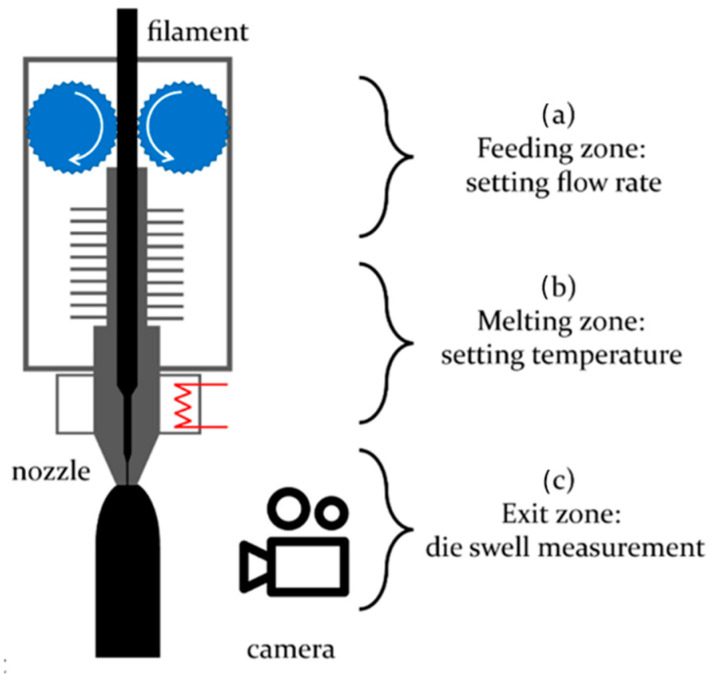
Die swelling during the FFF process. Reprinted from Ref. [25].

Die swelling is a common phenomenon influenced by shear thinning and viscoelasticity, taking place in the vicinity of the nozzle as shown in Figure 3.

Comminal et al. [22] focus on the flow regions near the die exits in their 2D simulations of extrudate swelling. The isothermal creeping flows of incompressible non-Newtonian fluids are governed by the conservation of mass and momentum, expressed in Equations (1) and (2). The researchers employed the Carreau–Yasuda fluid model to define the apparent viscosity, as specified in Equation (4) (*a* = 2, $\eta_{\infty }=0$).

(9) $$\eta (\dot{\gamma })=\eta_{0}{\left(1+{\left(\kappa \dot{\gamma }\right)}^{2}\right)}^{\frac{n-1}{2}}$$

They defined the degree of swelling in the extrudate as the as the ratio of the extrudate thickness *D_extr_* to the nozzle inner diameter *D_in_*:

(10) $$Sr=\frac{D_{extr}}{D_{in}}$$

**Figure 4:**
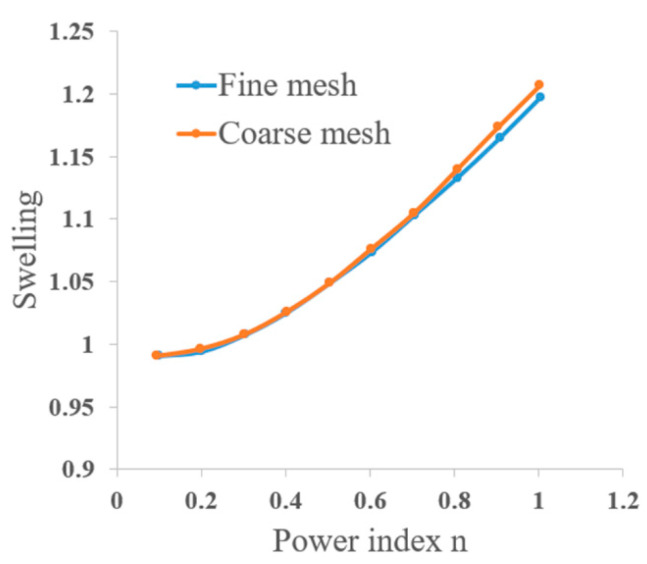
Extrudate swell of the power law fluid as a function of the power law index n [22].

By selecting very small values for *k* and very large values for $\eta_{0}$*,* the Carreau–Yasuda fluid model in Equation (9) can be reasonably converted to the power law. In their numerical model, the researchers measured the degree of swelling for different values of the power index *n*. The results are shown in Figure 4. As expected, shear thinning reduces the extrudate swelling.

Shadvar et al. [26] examined the behavior of an ABS melt polymer during the extrusion process of FFF and the die swell of the extruded polymer by empirical experiments and simulation using ANSYS Polyflow. They investigated the internal geometry of the nozzle via analytical simulation and finite element analysis to obtain pressure and velocity distributions of the material inside the extruder as well as the die swell of the extruded filament. In this study, the flow profile of the molten material inside the nozzle was achieved by solving the Navier–Stokes equations for continuity and momentum simultaneously. This system of equations describes the governing equation of viscous flows in the laminar state [27,28,29,30]. The researchers solved energy equations for non-isothermal flows throughout the flow domain and classified polymeric melts as non-Newtonian fluids and their temperature-dependent rheological properties:

(11) $$\eta =F(\dot{\gamma })H(T)$$

**Figure 5:**
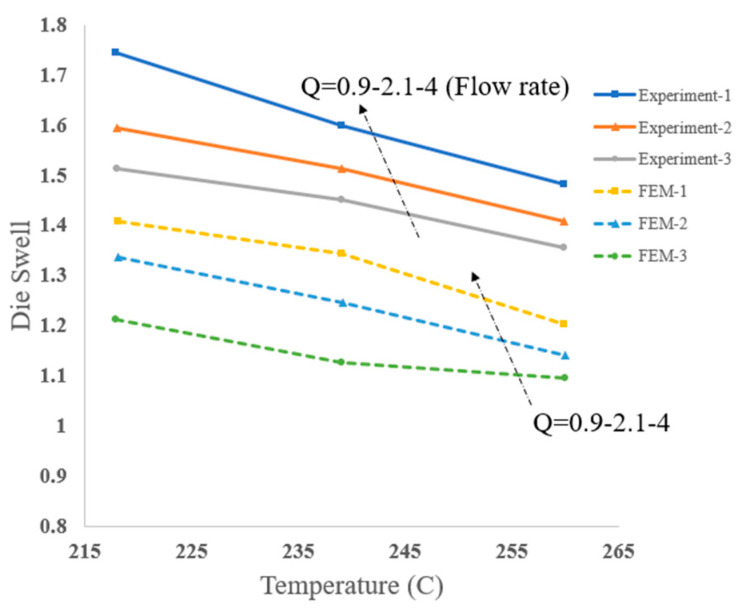
Die swell versus temperature at various flow rates Q (mm^3^/s) from experiments and FEA [26].

They defined the viscosity based on the power law (Equation (3)), and the temperature dependency of flow viscosity was defined based on the Arrhenius equation as

(12) $$H(T)=\exp\left(\alpha \left(\frac{1}{T}-\frac{1}{T_{\alpha }}\right)\right)$$

where α is the activation energy and is obtained by measuring the slope of the logarithmic plot of viscosity vs. the inverse of temperature within the limits of the intended shear rate. *T_a_* is the reference temperature at which *H* = 1. To reduce computational costs, an axisymmetric model is considered, as the geometry and loading are symmetric. Inside the liquefier and nozzle, the polymer behavior is generalized towards the Newtonian state. However, due to the high shear stress in the nozzle outlet section, the polymer behaves like a viscoelastic material. Thus, the researchers split their finite element simulation into two stages with two different non-Newtonian models for calculations. For simplicity in solving governing equations, they employed the following assumptions: laminar flow, steady-state regime, incompressible flow, no slip on the extruder walls, and no gravity force effects. It was also assumed that the flow rate at the inlet of the capillary was uniform with constant temperature and that the semi-molten ABS polymer was incompressible. The part of their simulation that includes the capillary of the nozzle and the extruded polymer was developed to investigate the die-swelling phenomenon. They considered a two-dimensional geometry model meshed by the quadrilateral element type. The authors carried out experiments to validate analytical and numerical simulations. The results revealed that a higher melt temperature and lower material flow rate resulted in less pressure drop inside the nozzle and less swelling of the extruded filament. The dependency of the degree of swelling on shear thinning has also been discussed by Comminal et al. [22]. Die swell as a function of temperature and for different flow rates is depicted in Figure 5. The results show that die swelling is highly dependent on extrusion temperature and flow rate. At higher extrusion temperatures and lower extrusion rates, the die swelling is minimized.

Ramanath et al. [30] used both mathematical modeling and FE analysis to investigate the influence of different parameters on the melt flow behavior of biocompatible materials such as poly-ε-caprolactone (PLC), which is frequently used in scaffolds. The FFF machine parameters that affect the melt flow behavior of polymers include build plate temperature, material flow rate throughout the nozzle, and extrusion temperature. The power law for non-Newtonian polymer melt is utilized to derive the mathematical model based on equation below:

(13) $$\dot{\gamma }=\emptyset \tau^{m}$$

where ∅ is fluidity, *τ* is shear stress, and *m* is the flow exponent. To measure the pressure drop at different sections of the nozzle, the authors considered the melt flow rate inside the nozzle as a steady-state isothermal process and used Bellini equations [31]. Bellini defines the pressure drop between two points located at a distance *L* inside the nozzle with diameter *r* as a function of the nozzle geometry and temperature-dependent viscosity, given in the Arrhenius equation (Equation (11)) as follows:

(14) $$\Delta P=2L{\left(\frac{v}{\emptyset }\right)}^{\frac{1}{m}}{\left(\frac{m+3}{r^{m+1}}\right)}^{\frac{1}{m}}\exp\left(\alpha \left(\frac{1}{T-T_{0}}-\frac{1}{T_{a}-T_{0}}\right)\right)$$

**Figure 6:**
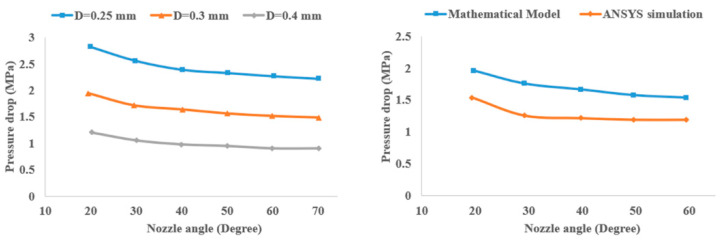
(**Left**) Pressure drop versus nozzle degree for different nozzle diameters; (**Right**) comparison between numerical model and simulation [30].

The relationship between force, pressure, and area is defined as *F* = Δ*P* × *A*. Based on the fact that the cross-sectional area of the nozzle and the force applied to the confined polymer melt by constantly rotating roller feeders are constant, any variation in pressure drop, which is common in real applications, will inevitably affect the part build. The authors used ANSYS FEA software to conduct FEA modeling in 2D and 3D. In order to have better control on mesh quality and sizing, they used the mapped meshing scheme in their study. For the 2D model, they used the Fluid141 element type, and for 3D analysis, they employed the Fluid142 element type. The physical and thermal properties of PCL were measured through experiments. The melting temperature of PCL was found to be 57.17 °C using a differential scanning calorimeter. A thermal conductivity of 0.203 W/m K was measured using a thermal conductivity tester. These properties were then used in the FEA model in ANSYS. Since there are many parameters to adjust during the FEA, such as nozzle diameter, nozzle angle, and inlet velocity, the authors conducted a parametric study using a Visual Basic graphical user interface to set up an automated FE analysis and perform the simulations more conveniently for different parameters. They studied three characteristics of the PLC melt flow, temperature distribution, pressure drop, and fluid velocity, using both the mathematical model and FE simulations. The comparison between the results for pressure drop obtained from both models is presented in Figure 6.

**Figure 7:**
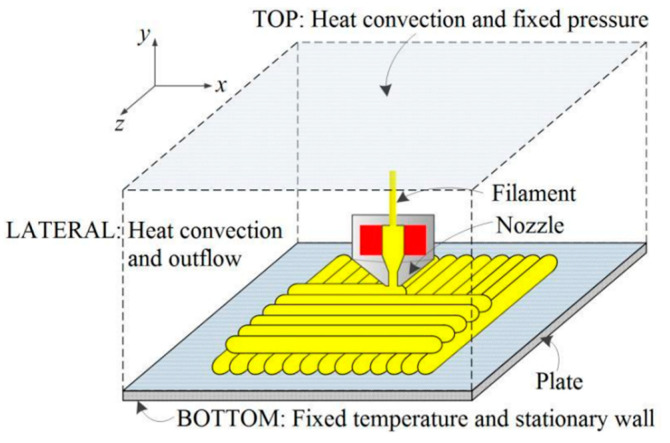
A schematic showing the computational domain. Reprinted from Ref. [33] with permission from EMERALD GROUP.

Xia et al. conducted a series of studies to develop a comprehensive methodology for the fully resolved numerical simulation of fusion deposition modeling. In the first study [32], they propose a numerical model to simulate the flow behavior of polymer melt in the FFF process. The hexahedral computational domain they considered in their study is shown in Figure 7.

The bottom section of the domain is considered adiabatic and at constant temperature to model the print bed, while the side walls and top portion of the domain are free surfaces, allowing air to freely circulate. The nozzle is modeled using a volume source that can move at a specified speed along a specific path. The dynamics of the problem are governed by Navier–Stokes equations. It is assumed that the viscosity of air is constant, while the viscosity of the polymer melt is a function of temperature and shear rate. Although the viscosity of polymer melt depends on pressure [34,35], the melt viscosity is considered independent of pressure since the melt pressure is close to the surrounding pressure and will not change after extrusion. Another frequently used model to express viscosity as a function of temperature and shear rate is the Cross-WLF model, defined as [21]

(15) $$\eta =\frac{\eta_{0}}{1+{\left(\frac{\eta_{0}\dot{\gamma }}{\tau }\right)}^{1-n}}$$

Suitable boundary conditions (BCs) are considered to implement the numerical method based on the finite volume/front tracking method initially introduced by Unverdi and Tryggvason [36]. For the bottom face of the model, a stationary wall boundary condition is applied for the velocity, and a fixed temperature of 40 °C is assigned to it. For the lateral sides, an outflow boundary condition is applied for velocity (∂u/∂n=0), where *n* is the unit vector normal to the wall. For the temperature boundary condition, a thermal convection is defined as follows:

(16) $$k\frac{\partial T}{\partial n}=h\left(T-T_{\infty }\right)$$

**Figure 8:**
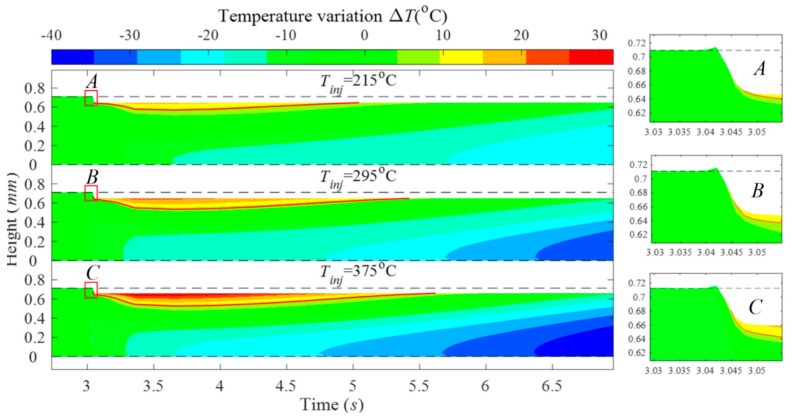
The evolution of temperature variation and deformation along a perpendicular line inside the bottom filament for three different injection temperatures. A, B, and C on the right frame show a closer view of the reheated zone corresponding to the left frame. Reprinted from [33] with permission from EMERALD GROUP.

For the top surface, however, an additional boundary condition of constant pressure is LAO defined. Employing the numerical model developed, researchers examine the role of print parameters such as print speed and extrusion temperature on melt flow. For example, the model clearly demonstrates that a higher print speed results in thinner layers, as expected, and that extruding material at higher temperatures creates greater temperature gradients in the surrounding material, as shown in Figure 8.

**Figure 9:**
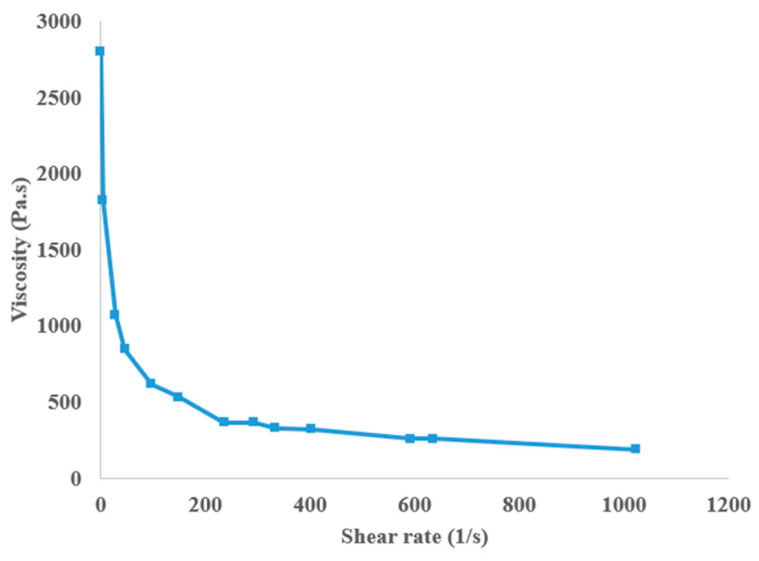
Viscosity versus shear rate for 10% filled iron–ABS composite at 270 °C [29].

Nikzad et al. [37] conducted 2D and 3D numerical analyses of the melt flow behavior of a representative ABS–iron composite through the 90 degree bent tube of the liquefier head in the fused deposition modeling process using ANSYS-FLOTRAN and the CFX Module of the ANSYS Workbench software. They investigated main flow parameters, including temperature, velocity, and pressure drop, in their simulations. The composite polymer they used was ABS plastic containing a 10% volume fraction of iron powders with an average particle size of 45 μm and a specific gravity of 7.88 g/cm^3^. By conducting experiments, they measured the physical and rheological properties of the developed composite and then used these experimental values in ANSYS software to investigate the melt flow behavior. Because of the non-Newtonian nature of the flow, viscosity was defined as a function of a power law. For the composite ABS–iron polymer, the value of K and the power law index *n* were calculated as 682.33 Pa·s and 0.4, respectively (see Figure 9).

The Ansys software provides users with pressure drop, velocity, and temperature profile data of the flow along the melt flow channel. Numerical results were also verified using a power law model suitable for non-Newtonian flows. In order to ensure that the simulation results were independent of mesh characteristics in 2D analysis, the researchers used both mapped and free meshing schemes. For 3D analysis, on the other hand, the CFX mesh was utilized, employing a combination of tetrahedral, pyramidal, and prism- shaped elements to achieve the optimum result. The total number of the finest mesh applied was 371,139. The authors predicted the velocity profile of the composite polymer flow inside the nozzle. In the middle of the nozzle, the flow velocity was maximum, while near the nozzle walls, the velocity was zero due to the no-slip boundary conditions applied. As the molten composite was pushed down from the start point of the nozzle by the solid filament, the pressure drop increased and reached its maximum value at the nozzle exit point. The large pressure drop at the nozzle exit could not be compensated for by the steady feeding of filament by roller feeders [30]. As discussed before, this pressure drop, if not compensated, will negatively affect the quality of the structure of printed parts. One possible solution to compensate for this pressure drop is to install a force feedback control system on printers to check the flow pressure in real time and change the flow rate accordingly.

**Figure 10:**
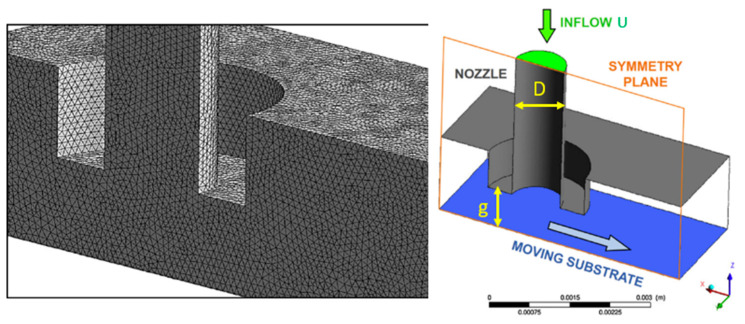
Geometry of the numerical model (**right**) and details of the tetrahedral mesh (**left**). D is the nozzle diameter, g represents the gap between the nozzle and the print bed, and V is the print speed. Reprinted from Ref. [38] with permission from Elsevier.

Comminal et al. [38] numerically investigated the influence of print parameters and processing conditions on the cross-sectional profile of a strand printed by material extrusion additive manufacturing and then validated their numerical model empirically [39]. They used the CFD software ANSYS^®^ Fluent R18.2 to model material deposition in the material extrusion process. Their numerical model simulated the flow of the material in its molten state in the region between the nozzle and printing bed. For simplicity, the authors modeled the extrusion nozzle as a circular cylinder with diameter D. Even though they considered the nozzle stationary and the substrate moving at a constant speed in their model, in a steady-state extrusion, both possible configurations—moving nozzle with fixed substrate and fixed nozzle with moving substrate—can be used interchangeably. Another simplification assumption that makes their numerical model more efficient in terms of time and CPU usage is that they considered only half of the physical domain in the simulation. This is because the material flow through the nozzle to the substrate is symmetrical with respect to the symmetry plane of the nozzle and parallel to the print direction. The simulation domain, boundary conditions, and mesh are presented in Figure 10.

As a realistic boundary condition, the authors considered slip-free contact on the substrate and at the walls of the nozzle because perfect bonding between the material and substrate was assumed. The rest of the boundaries of the model were prescribed as outlet boundary conditions since the material is free to exit from these boundaries. As previously mentioned, simplification is an inevitable part of numerical analysis. The authors made several assumptions to simplify flow simulation during material extrusion. First, they assumed that the deposition flow is isothermal. Second, they considered inertial effects negligible, indicating that the material extrusion occurs in a creeping flow regime and that the actual values of the density *ρ* and viscosity μ have no effect on the simulation results. Third, the fluid flow speed was assumed to be constant. Since the dynamic viscosity of the polymer melt has no influence on the strand shape, the material was modeled using a linear constitutive behavior following Newtonian fluid behavior with constant viscosity η, defined as

(17) $$\tau =\eta \dot{\gamma }$$

**Figure 11:**
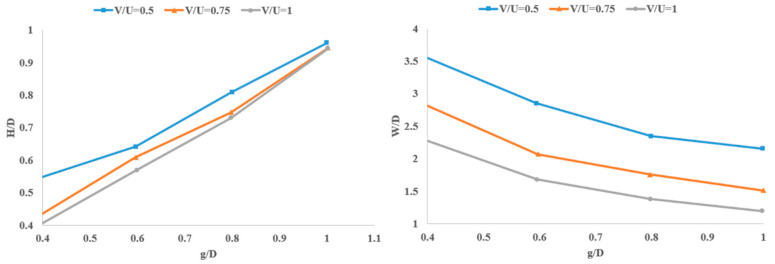
Strand thickness (**left**) and strand width (**right**) as functions of print parameters; H/D is the relative bead thickness, W/D is the relative bead width, V/U is the relative nozzle movement (w.r.t. the print bed), and g/D represents the relative gap between the nozzle and the print bed. Reprinted from Ref. [38] with permission from Elsevier.

As the extruded beads of material in the extrusion process are round and cylindrical in shape, tetrahedral elements are the best candidates and are frequently used in simulations [40]. The authors used tetrahedral elements for meshing the model in their simulation, as shown in Figure 10. They employed a coupled pressure–velocity solver with implicit time-marching discretization to solve the conservation of mass and momentum equations. They investigated the effect of print parameters such as print speed and the gap between the nozzle and substrate on the aspect ratio and cross-sectional area of the printed material (See Figure 11).

**Figure 12:**
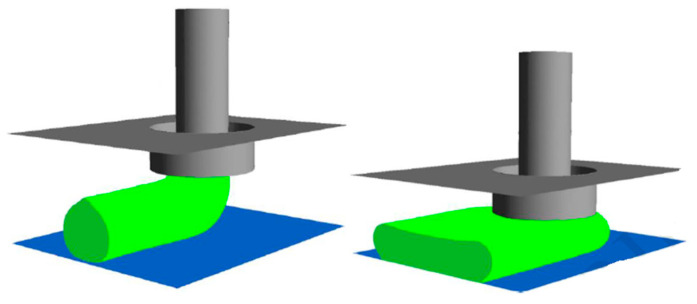
Perspective views of the simulated strand deposition for a large, normalized gap height g/D = 1.2 at a high velocity ratio V/U = 1 (**left**) and for a small, normalized gap height g/D = 0.6 at a low velocity ratio V/U = 0.5 (**right**). Reprinted from Ref. [39] with permission from Elsevier.

In Figure 11, *U* is the melt flow speed inside the nozzle, *V* is the relative movement of the nozzle with respect to the substrate, g is the gap space, *D* is the nozzle diameter, and *H* and *W* are the height and width of the material strands. As expected, high print speeds and small gaps result in small aspect ratios of the material strands, while slow printing with small gaps between the nozzle and the build plate flattens the material strands and results in larger aspect ratios, as clearly shown in Figure 12 [39]. The authors finally observed that numerical simulations can predict different morphologies of the deposited strand by comparing the results with experimental data.

**Figure 13:**
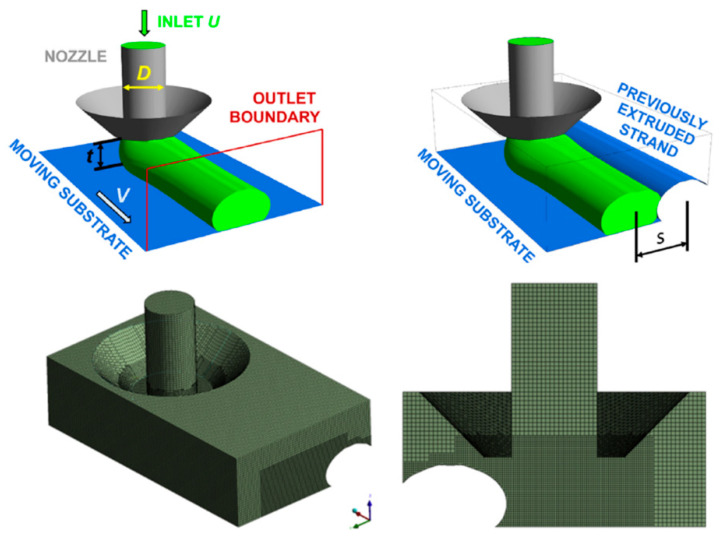
Geometry of the computational domain (**top**); meshed model (**bottom**); *t* is the nozzle distance from the print bed, V is the relative printing bed movement, and *s* is the distance between two adjacent beads (hatch distance). Reprinted from Ref. [41] with permission from Elsevier.

Serdeczny et al., in another study, presented a numerical technique to simulate the deposition of multiple layers of parallel strands resulting in a representative volume element of the meso-structure [41]. The extrusion and deposition of the polymer beads are simulated using a CFD model [42]. The authors considered deposition governed by the Navier–Stokes equations, which account for the conservation of mass and momentum, similar to their study in Ref. [39]. Some assumptions considered in their proposed model to simplify the simulation process are as follows: pure adhesion between the recently deposited molten material and the previously deposited substrate is considered; the velocity of the molten material flowing through the nozzle is constant; the density of the material remains constant during phase and state changes; and since polymers typically have a low thermal conductivity, they assume that the reheating of the previously deposited material by the newly extruded melt does not influence its shape. However, the presence of the already solidified substrate affects the deposition of subsequently extruded material, which affects the formation of the meso-structure and the degree of crystallinity [43,44]. The authors used ANSYS Fluent R18.2 to simulate flows in their numerical model. The computational domains were discretized with a structured Cartesian cut-cell mesh, and the governing equations of the flow were discretized using a collocated finite volume method. The geometry of the computational model, including the BC and meshed part, is presented in Figure 13.

**Figure 14:**
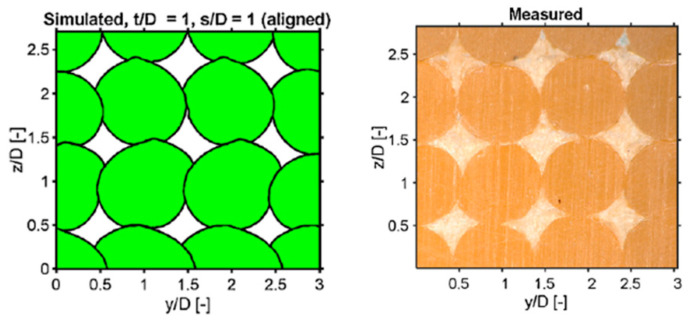
Comparison between simulated (**left**) and measured (**right**) strand profiles. Reprinted from Ref. [41] with permission from Elsevier.

The implicit coupled pressure–velocity scheme was employed to solve for the transient-state fluid flow of the molten (semi-solid) material. The authors simulated the free-surface flow as a two-phase flow of the melt and the surrounding atmosphere, and the free surface of the molten plastic was tracked with the coupled level-set and volume-of-fluid methods [45]. The simulations implemented by the authors provided thorough and detailed predictions about the porosity of the printed geometry, the inter-layer and intra-layer bond line densities, and the surface roughness of the structures. The comparison between numerical simulations and experimental results, obtained from microscopic imaging of polished strand cross sections, is shown in Figure 14.

The authors investigated the effect of different print parameters on porosity, strand morphology, bond line density, and surface roughness. A summary of the results obtained from their study is as follows: the porosity of printed parts increased with the strand-to-strand distance and the layer thickness; the skewed deposition configuration resulted in lower porosities compared to the aligned configuration; the horizontal surface roughness was reduced by decreasing the strand-to-strand distance, which forced the melt flow to fill the intra-layer cavities; and the vertical surface roughness was weakly dependent on the strand-to-strand distance.

The numerical models presented in this section for simulating melt flow behavior in the FFF process include, but not limited to, the Navier–Stokes equations for incompressible, non-Newtonian, creeping flow; several rheological models such as the power law, Carreau–Yasuda, Cross-WLF models to describe shear and normal viscosity; and different finite element and finite volume methods, primarily implemented in ANSYS software for 2D and 3D problems. Each model is able to capture essential aspects of melt flow, such as shear thinning, viscoelasticity, die swelling, and pressure drops throughout the nozzle. Obviously, the strengths of these models lie in their comprehensive flow descriptions, validation against experimental data, detailed analysis of involved parameters, and robust numerical methodologies, ensuring precise simulations. However, their accuracy will be challenged by simplifying assumptions that may not fully consider real-world complexities, and they may require significant computational resources, particularly for large-scale additive manufacturing; have limited generalization due to specific assumptions made throughout analysis; and be highly dependent on mesh quality. Overall, even though these proposed models provide valuable insights into the FFF process, their accuracy in predicting requested results is often balanced against computational cost and necessary assumptions to simplify complex physical phenomena.

## 3. Solidification and Crystallization Kinetics

During the FFF process, the molten polymer cools very quickly from a molten to a fully solidified state. Semi-crystalline polymers like polyetherketonketone (PEKK), polyetheretherketone (PEEK), polyphenylene sulfide (PPS), and polyethylenimine (PEI) partly crystallize when they are cooled from the melt [46]. Material crystallization is an exothermic process during which the polymer chains locally fold into dense arrangements called crystals. This crystal formation process finally results in the shrinkage and deformation of the material. The nucleation and growth of crystal chains are the governing mechanisms controlling the degree of crystallinity [44].

Crystallization time is a key parameter that determines the micro-structure, meso-structure, and macro-structure of additively manufactured components made of advanced semi-crystalline polymers. Strong bonding between two layers is possible only when deposition occurs on top of amorphous material, which is not usually the case for the FFF process, where the deposited material crystallizes quickly and the next layer is placed on crystallized beads of material. Crystallized material forms a thermally resistant shield for a newly printed layer immediately after deposition, which shows a significantly lower tendency to melt compared to amorphous material [46]. This results in weak inter-layer bonding and severe anisotropy in FFF-printed parts.

The most frequently used crystallization kinetics model is probably the one proposed by Avrami [47]. Avrami’s simplified model for isothermal crystallization is defined as [44]

(18) $$X_{vc}=1-\exp(-E)$$

where $X_{vc}$ is the crystal volume fraction, and *E* is called the extended volume fraction. When a semi-crystalline polymer melt is cooled down into its crystallization temperature range, crystallization starts around discrete points called “nuclei”, and the crystals grow around nuclei to form what are referred to as “spherulites” [48,49]. In the case of isothermal crystallization, the spherulite growth rate is assumed to be constant. Therefore, the extended volume fraction *E* can be defined as [44]

(19) $$E=kt^{n}$$

where *n* is the Avrami exponent, and *k* is the Avrami isothermal crystallization rate constant. Another Avrami-type model frequently used for non-isothermal crystallization was developed by Ozawa [50]. Considering a constant cooling rate (β) and the temperature dependency of nucleation and growth rate, the common form of the Ozawa model can be implemented as

(20) $$X_{vc}=X_{vc\infty }\left[1-\exp\left(k(T)/\beta^{n}\right)\right]$$

In Equation (20), $X_{vc\infty }$ is the maximum crystallinity that the material can achieve when fully solidified, *k* is the temperature-dependent crystallization rate, and *n* is the Ozawa exponent. Nakamura and Watanabe [51] developed another non-isothermal crystallization kinetics model derived from the Avrami model.

(21) $$X_{vc}=1-\exp{\left[1-\int_{0}^{t}K(T)dt\right]}^{n}$$

where *K*(*T*) is the non-isothermal crystallization rate, which is a function of temperature and crystallization half-time, described fully in Ref. [52]. The crystallization half-time corresponds to a crystallized volume fraction of 0.5 [53,54].

Brahmia et al. [48] used the COMSOL Multiphysics software package to study the coupling of heat transfer and crystallization kinetics during the solidification of polypropylene in injection molding, which shares many features with extrusion-based additive manufacturing processes like FFF. In order to simulate the injection molding process, they placed a plate of polypropylene between two metallic plates and used a general energy equation to define heat transfer between metal plates and polymeric plates. They employed isothermal and non-isothermal DSC experiments to characterize the material of interest and simulated the crystallization kinetics using the Avrami and Nakamura models for isothermal and non-isothermal crystallization, respectively. They solved Nakamura crystallization kinetics using the diffusion equation in COMSOL Multiphysics and MATLAB software using a finite differences scheme to discretize the equations. A good agreement was observed between the results from COMSOL and the data from MATLAB simulations.

The authors implemented several simulations to investigate the effect of different parameters such as the initial temperature of the polymer plate and the cooling rate on the evolution of crystallinity. They observed that the crystallization temperature range shifts 10 degrees for different cooling rates. Consequently, the crystallization temperature range for semi-crystalline polymers is not necessarily fixed and depends on the cooling rate. Additionally, they concluded that the material near the mold walls crystallizes faster compared to that in the internal section of the polymer plate far from the mold surface. This phenomenon is attributed to the perfect contact between the metal plates and polymer defined in the simulation, in which heat is dissipated more efficiently from the metal plates due to higher thermal conductivity. Furthermore, crystallization begins approximately at the same temperature at the mold/polymer interface and the polymer core and continues over the same temperature range. However, the slope of the crystallization graph at the interface is steeper, indicating that crystallization is completed more rapidly at the interface compared to the core area.

Brenken et al. [55] developed a numerical tool to simulate the heat transfer and the crystallization behavior of FFF-printed components. They modeled the cross sections of fiber-reinforced semi-crystalline thermoplastic polymers (CF PPS) in a 2D space using the multiphysics package COMSOL. They considered a transient heat transfer analysis coupled with a non-isothermal crystallization kinetics model to dynamically predict the temperature and crystallinity profile during the FFF process. In order to obtain the thermal cooling history during the FFF printing process, they implemented a numerical heat transfer analysis using COMSOL, considering the governing differential heat equation as described in Equation (22):

(22) $$\rho C_{p}\frac{\partial T}{\partial t}-\nabla \cdot (k\nabla T)=Q$$

where *ρ* is the density, *C_p_* the heat capacity, and *k* is the thermal conductivity implemented as a tensor since the material used in their study is anisotropic. The term *Q* represents external heat sources used to couple the crystallization kinetics analysis. Unlike many studies that neglect the external heat source as a simplification assumption, the authors considered it in their study because polymer crystallization is an exothermic process, which releases heat, and therefore, the thermal analysis is strongly coupled to the crystallization kinetics analysis. Therefore, for a semi-crystalline polymer, the heat generation term *Q* is present [55]. It can be formulated based on the crystalline volume fraction and the heat of fusion *H_f_* as [56]

(23) $$Q=\rho H_{f}\frac{dX_{vc}}{dt}$$

The authors used suitable boundary conditions as follows: the bed temperature was imposed on the bottom edges of the lowest layer in contact with the printing bed; free and forced convective heat flux boundary conditions were introduced for the outer edges of the cross-section geometry in direct contact with the surrounding atmosphere; and for closed internal surfaces, a thermal insulation boundary condition was chosen. Applying accurate boundary conditions is probably one of the most challenging parts of FEA, as it is almost impossible to mimic the real environment using available tools. Therefore, some simplifications are inevitable in FEA. Since the beads extend in the stacking direction in the real FFF-printed part, the heat transfer that occurs between the inner surfaces was assumed to be negligible in their analysis. The authors implemented a non-isothermal crystallization kinetics model proposed by Velisaris and Seferis in COMSOL using distributed ODE interfaces for numerical analysis. This model was initially developed to characterize the crystallization kinetics of polyether ether ketone (PEEK); however, it has successfully been employed to simulate the crystallization kinetics of other fiber-reinforced materials as well.

An efficient numerical model of the improved material extrusion (or FFF) process was established by Jun et al. [27] to examine the temperature profile and 3D solidified morphology. They considered the dependency relationship among the forming temperature, shear rate, and thermo-physical properties of the building material, such as viscosity and surface tension, in their proposed model. The dynamic flow field inside the molten material flowing through the nozzle was resolved using Navier–Stokes and energy equations [28]. They considered the viscosity a function of temperature and shear rate in their improved FFF computational model. The ABS melt, as the forming material used in the simulations, was assumed to be a non-Newtonian fluid, and the Carreau viscous model [29] was used for the simulation of the non-isothermal flow, as opposed to Ref. [39], where material flow was assumed to be isothermal. To examine the accuracy of their numerical model, they compared the temperature information and the solidified morphology obtained from their proposed model with experimental results. The numerical method based on the semi-implicit pressure-linked equation (SIMPLE) algorithm was adopted to simulate the improved material extrusion process. In order to simulate the free surface, they employed the volume-of-fluid (VOF) method with piecewise linear interface construction (PLIC). By considering two assumptions of incompressible and laminar flow, the continuum governing equations for macroscopic transport in a Cartesian coordinate system were used by the authors.

The solidification and crystallization kinetics of semi-crystalline polymers during the FFF process significantly affect the micro-, meso-, and macro-structures of additively manufactured parts. Semi-crystalline polymers such as polyetherketonketone (PEKK), polyetheretherketone (PEEK), polyphenylene sulfide (PPS), and polyethylenimine (PEI) crystallize rapidly upon cooling, resulting in the shrinkage and consequent deformation and warpage of the final components. The nucleation and growth of crystal chains are the primary mechanisms that govern the degree of crystallinity. Avrami-type numerical models, along with variations like the Ozawa and Nakamura models, are commonly used to describe crystallization kinetics. These models account for isothermal and non-isothermal crystallization behavior, considering factors such as cooling rate and temperature dependency. Finite element simulations using software packages like COMSOL Multiphysics and MATLAB are widely used to validate these models. The aforementioned numerical tools developed to simulate heat transfer and crystallization in FFF components incorporate transient heat transfer analysis and non-isothermal crystallization kinetics models. The accuracy of these models and simulations depends highly on applying suitable and realistic boundary conditions and accurately representing external heat sources, as polymer crystallization is an exothermic process. In summary, these models are considered potential tools for analyzing the crystallization behavior of semi-crystalline polymers in FFF, despite the challenges in reflecting real-world conditions and the requirement for simplification assumptions in finite element analysis.

## 4. Bonding Efficiency

**Figure 15:**
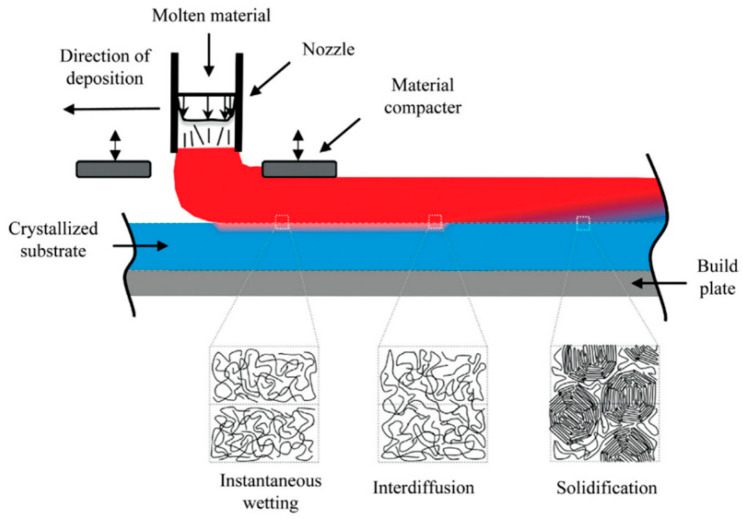
Schematic representation of the fusion bonding of polymers. Reprinted from Ref. [43] with permission from Elsevier.

FFF-manufactured components intended to be used for structural applications and industries still face challenges, particularly the poor mechanical properties in the layer-stacking direction caused by weak inter-layer bonding of polymeric materials. The deposition of a molten polymer on an already-cooled substrate during the FFF process causes residual thermal stresses to initiate in inter-layer areas. These residual stresses, combined with comparatively weak inter-layer properties, result in what is commonly known as debonding (Figure 15). Therefore, it is of great importance to understand the effect of different parameters on bonding efficiency by applying available models and to use these models to develop new methods in order to better address the poor inter-layer bonding challenge facing FFF-manufactured polymeric parts.

The isothermal healing of a polymer interface via molecular interdiffusion is often described using the reptation theory, which models the motion of individual linear polymer chains in an amorphous bulk [57]. The degree of healing may be defined as the ratio of the instantaneous interfacial bond strength (*σ*) to the ultimate bond strength ($\sigma_{\infty }$) for fully solidified material as [58,59]

(24) $$D_{h}=\frac{\sigma }{\sigma_{\infty }}$$

For isothermal conditions, the degree of healing can be formulated as [60]

(25) $$D_{h}={\left(\frac{t}{t_{R}}\right)}^{1/4}$$

where *t* is the time, and *t_R_* is the reptation time, which is a function of temperature and can be expressed using an Arrhenius-type equation or any other experimentally derived expression. Bastien and Gillespie [61] extended the isothermal healing model expressed in Equation (25) and derived a non-isothermal fusion bonding model for amorphous thermoplastic laminates.

Basgul et al. [62] proposed a heat transfer-based non-isothermal healing model for the interfacial bonding strength of fused filament-fabricated PEEK. They developed a one-dimensional heat transfer model to compute the intra-layer and inter-layer temperature distribution in the FFF process of PEEK. Then, they coupled their heat transfer model with a non-isothermal healing model to predict the inter-layer strength through the thickness of the printed part. The non-isothermal degree of healing utilized in their study was developed initially by Yang and Pitchumani [57] as

(26) $$D_{h}=\frac{\sigma }{\sigma_{\infty }}={\left[\int_{0}^{t}\frac{1}{t_{W}(T)}d\tau \right]}^{\frac{1}{4}}$$

where $t_{w}$ is the temperature-dependent welding time. The authors conducted a parametric study on the effect of different print parameters, including the printing bed temperature, extrusion temperature, and chamber temperatures, on the degree of healing. Based on the results obtained from their model, which are in agreement with experimental results, the upper layers with respect to the print bed exhibited higher temperatures and therefore higher degrees of healing compared to the lower layers. Increasing the print bed temperature enhanced the healing of the layers. The nozzle temperature had the most significant effect on layer healing, and under certain nozzle temperatures, the degree of bonding never reached 100%. Another parameter that affected the inter-layer healing was the chamber temperature. The effect of this parameter was more obvious on the top layers than on the bottom layers close to the printing bed.

Barocio et al. [63] implemented a phenomenological model for the fusion bonding of semi-crystalline polymer matrix composites. The strength of their inter-layer bonding model is the capability of coupling the interdiffusion of polymer chains with the evolution of polymer crystallinity. They used the reptation theory of polymer dynamics to capture the interdiffusion of polymer chains, and the evolution of crystallinity was modeled using phenomenological crystallization kinetics and crystal melting dynamics [44]. The authors extended the healing model developed by Yang and Pitchumani [57] to describe the non-isothermal evolution of time, temperature, and crystallinity-dependent interdiffusion. In Yang’s model mentioned in Equation (26), the integration time domain is limited by the development of crystallinity. To capture the effect of crystallization on interdiffusion, the integral is evaluated only in the time domain, wherein the degree of crystallinity *X_vc_* is less than the critical degree of crystallinity *X_crit_*. *X_crit_* is defined as the degree of crystallinity corresponding to the termination of polymer chain interdiffusion [63]. As previously stated, the welding time $t_{w}$ may be expressed by the Arrhenius equation. The authors used such an equation to derive the welding time as

(27) $$t_{w}(T)=A\cdot \exp\left(\frac{E_{A}}{RT}\right)$$

where *A* is a material constant, *E_A_* is the activation energy, and *R* is the universal gas constant. They used Abaqus software to implement their numerical model. Since temperature and crystallinity are calculated inside the UMATHT user subroutine throughout the analysis, the degree of bonding is also implemented in the UMATHT, as it is a function of the temperature and degree of crystallinity. Finally, they used their predictive healing model to develop methodologies to determine the critical strain energy release rate and fracture toughness of the inter-layer interface as a potential area for crack initiation and propagation.

**Figure 16:**
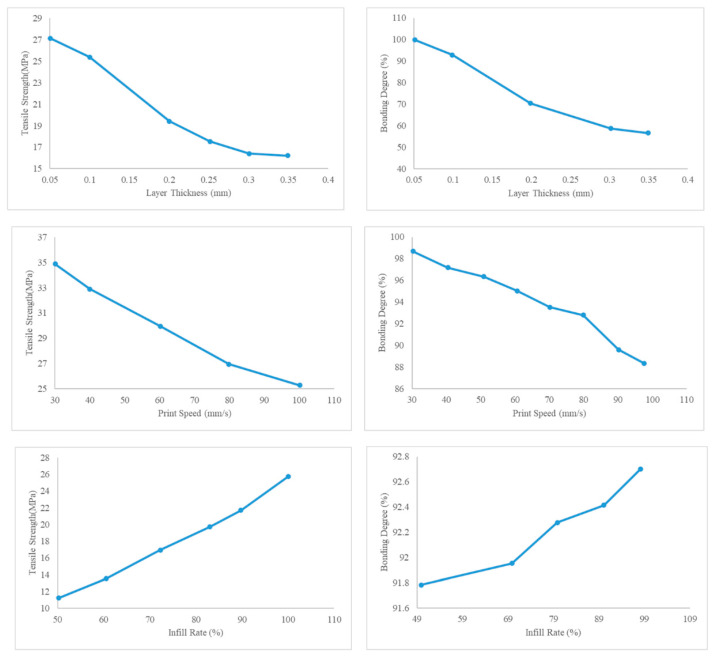
The effect of different print parameters on tensile strength ((**left**) column) and bonding degree ((**right**) column) [64].

Li et al. [64] proposed a numerical model to quantitatively describe the relationship between different print parameters such as print speed, infill rate, and layer thickness on degree of bonding and the effect of the degree of bonding on the mechanical properties of FFF-printed parts made of PLA. To implement the bonding behavior of the material, they utilized a model originally developed by Wool et al. [65]. A part of their results on the influence of print parameters on the degree of bonding and tensile strength of the material is shown in Figure 16.

As shown in the figures, the tensile strength is closely related to the interface bonding degree. Since the bonding degree is determined by heat transition, material properties such as tensile strength are affected by heat transition as well. Based on the data obtained from their model and experiments, layer thickness has the greatest effect on the bonding strength; thick layers lead to weaker inter-layer bonding and a lower tensile strength of the printed part. The next parameter that affects the bonding and tensile strength of the printed parts is printing speed. Slow printing results in better inter-layer adhesion and stronger parts. Among these parameters, infill rate has the weakest effect on the bonding degree and material strength.

Bellehumeur et al. [66] modeled the bond formation between polymer filaments in the FFF process and determined the effect of different print parameters on bonding strength in an ABS P400 polymer (Stratasys Inc., Eden Prairie, MN, USA; Rehovot, Israel). They performed an ANOVA analysis with a significance level of 0.1 and utilized the commercial software MINITAB^®^ for statistical analysis. In their analysis, they measured the effects of extrusion temperature, envelope temperature, and the convective heat transfer coefficient on neck growth, which is an indicator of bonding. Their results showed that, for example, the extrusion temperature has a greater effect on neck growth during the material cooling process compared to envelope temperature.

The improvement of FFF-manufactured component functionality through enhanced bonding efficiency has been investigated both numerically and experimentally and has proven to be an effective technique for enhancing the reliability of industrial part production. Yu et al. [67] proposed a methodology to improve the mechanical properties of PLA FFF-printed parts using a 3D printer with a second-generation auxiliary heating plate attached to the extruder head. This auxiliary heating plate preheats the previously deposited layer and enhances inter-layer adhesion. With this treatment, the tensile strength of the FFF-printed PLA part increased from 38.4 MPa to 63.6 MPa, and the degree of mechanical anisotropy decreased from 0.51 to 0.22. Kumar et al. [68] experimentally demonstrated that increasing the layer thickness reduces the tensile strength of FFF-printed parts, mainly because thick layers result in poor layer adhesion. Fusing multiple layers together by reducing layer thickness improves bonding strength, achieving closely packed inter-layers, low porosity, and high density.

Sabyrov et al. [69] used a 5 W, 450 nm diode laser for the localized heating of the pre-deposition layer in order to improve the mechanical properties of PLA parts by improving layer adhesion. By controlling laser power, the layer interface temperature reached a critical point at which the bonding diffusion between layers increased to the maximum level. They found that using the optimum laser power of 2.84 W resulted in a 10.16% increase in the ultimate tensile strength of printed parts. Lyu et al. [70] introduced a chemical bonding method based on the physical welding of polymers to enhance the performance of 3D-printed complex bi-material products. They set up the Diels-Alder (D-A) reversible thermoset printing process. Based on the D-A reversible reaction mechanism, they introduced furanized PLA, furfurylamine, and bismaleimide into the matrix of a PLA blend with a compatibilizer to construct a reversible dynamic covalent bond. The tensile testing of printed parts demonstrated that increasing D-A bonds significantly improved both tensile strength and inter-layer bonding. The tensile strength of 3D printed bi-material parts increased by 408% after introducing D-A reversible covalent bonds.

Yao et al. [71] proposed a layer-wise, in-process laser treatment technique to improve inter-layer bonding efficiency in the FFF process of both pure and composite polymers. The laser-assisted process selectively treats pre-deposition layers, significantly improving the layer-to-layer bonding. The results showed significant increases of over 20% in the tensile strength and of 10–20% in the bending strength of printed parts. Li et al. [72] proposed a new method to enhance the interfacial bonding strength of PEEK. They utilized the interface plasticizing effect of benzene derivatives obtained from the thermal pyrolysis of trisilanolphenyl polyhedral oligomeric silsequioxane (POSS). Their results show that using POSS/PEEK instead of PEEK achieved a bonding strength of up to 82.9 MPa for filaments (intra-layer) and 59.8 MPa for inter-layer bonding strength. Improved inter-layer bonding is particularly important in the FFF process of parts made of multiple materials [11,73].

FFF-manufactured components for heavy-duty applications still face challenges stemming from poor mechanical properties in the stacking direction due to the weak inter-layer bonding of polymeric materials. The deposition of a molten polymer on a low-temperature substrate or on previously cooled layers during the FFF process causes residual thermal stresses in the inter-layer areas to grow, which can ultimately lead to debonding. Understanding the effect of various parameters on bonding efficiency is of great importance for tackling this problem. Among the leading theories in this area is the reptation theory, which describes isothermal healing via molecular interdiffusion. It defines the degree of healing as the ratio of instantaneous interfacial bond strength to ultimate bond strength. Non-isothermal fusion bonding models, such as those proposed by Bastien and Gillespie and extended by Basgul et al., combine heat transfer with inter-layer bonding strength. These models consider temperature distribution and the non-isothermal degree of healing influenced by print parameters such as bed temperature, extrusion temperature, and chamber temperature. Abaqus and ANOVA are frequently used to prepare methodologies and numerical models to quantify the relationship between print parameters and bonding efficiency. These studies reveal that layer thickness, print speed, and infill rate affect tensile strength and bonding degree, with extrusion temperature having a major impact.

Aside from numerical models and pure simulations, various experimental techniques, including auxiliary heating plates, laser treatments, and chemical bonding, have proven effective in boosting inter-layer bonding efficiency. Localized heating with a diode laser helps to achieve a considerable increase in the tensile strength of printed parts. Chemical bonding with reversible Diels–Alder reactions can significantly improve tensile strength and bonding efficiency. Laser treatment has also been shown to improve bonding efficiency, resulting in increased tensile and bending strengths. Improving inter-layer bonding is critical for the FFF process, especially in multi-material FFF applications, as it enhances the reliability of heavy-duty industrial components.

## 5. Thermal Analysis

**Figure 17:**
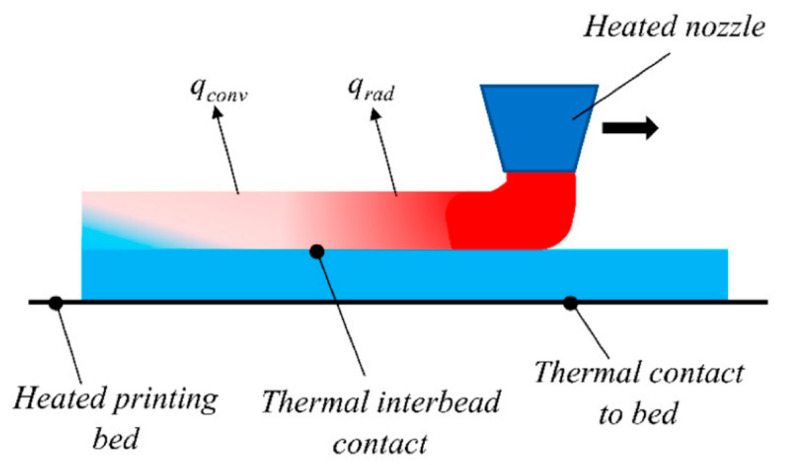
Heat transfer during the FFF process. Reprinted from [74].

In the FFF process, a hot molten polymer is continuously deposited layer by layer on a platform or on the previously deposited material to build a 3D part. At the same time, the previously deposited material cools down from its initial molten state at the melting point to room temperature [44]. Figure 17 depicts the general thermal configuration of the FFF process, considering all the possible boundary conditions. These include thermal contact between the printing bed and the first layer, which maintains a constant temperature and resistance between roads of material, and convective heat transfer between the deposited material and the surrounding atmosphere.

The thermal problem of the FFF process can be fully described by combining the first law of thermodynamics and Fourier’s law as [55]

(28) $$\nabla \cdot \left(k\nabla T\right)+\dot{Q}=\rho C\frac{dT}{dt}$$

where *ρ* is the density of the material, *C* is the specific heat capacity, and *k* is the thermal conductivity. This equation is derived from the conservation of energy, the first law of thermodynamics, which states that the rate of heat entering a control volume ϑ through the bounding surface plus the rate of energy generation in ϑ equals the rate of energy storage in ϑ [44]. *Q* is generated heat, also referred to as the external heat source. Since crystallization occurs in the FFF process of semi-crystalline polymers, this term in the equation is not negligible. Brenken [44] defines this generated heat as a function of the crystallization latent heat *H* and crystallization volume fraction *X:*

(29) $$\dot{Q}=\rho H\frac{dX}{dt}$$

where

(30) H=∫ρC(T)dT

This general formulation for heat transfer in the FFF process can be simplified by considering simplification assumptions. For example, one may consider no heat generation in the case that the material used is an amorphous polymer. Additionally, the material can be considered thermally isotropic, which reduces the conductivity tensor from three different conductivities (in three different directions of the Cartesian coordinate system) to a single value as a function of temperature, etc.

Jensen et al. [75] proposed a simplified finite volume model to describe the heat transfer phenomenon in thermoplastic and thermoset polymers. The authors made several assumptions to simplify the proposed model. First, they considered their model in two-dimensional space and neglected the print direction in their analysis. Second, they assumed that the material deposition is a sequential addition of solid material and that conduction heat transfer is the only dominant heat transfer phenomenon. Then, heat transfer can be simply described using Fourier’s law and the first law of thermodynamics, as in Refs. [76,77]. To implement these equations in the simulations, they used finite volume discretization and applied a fully implicit solution. The authors utilized a 2D MATLAB code to describe the finite volume method of thermal analysis. They verified their proposed model using experimental data, and a good agreement was obtained.

Zhou et al. [77] developed a fully 3D thermal analysis for the fused deposition modeling of polymers (ABS) in ANSYS APDL. Thermal analysis of the FFF process considered in their study was 3D and nonlinear, without a volume heat source, derived from Equation (28) as

(31) $$\frac{\partial }{\partial x}\left(k_{x}\frac{\partial T}{\partial x}\right)+\frac{\partial }{\partial y}\left(k_{y}\frac{\partial T}{\partial y}\right)+\frac{\partial }{\partial z}\left(k_{z}\frac{\partial T}{\partial z}\right)=\rho C\frac{\partial T}{\partial t}$$

**Figure 18:**
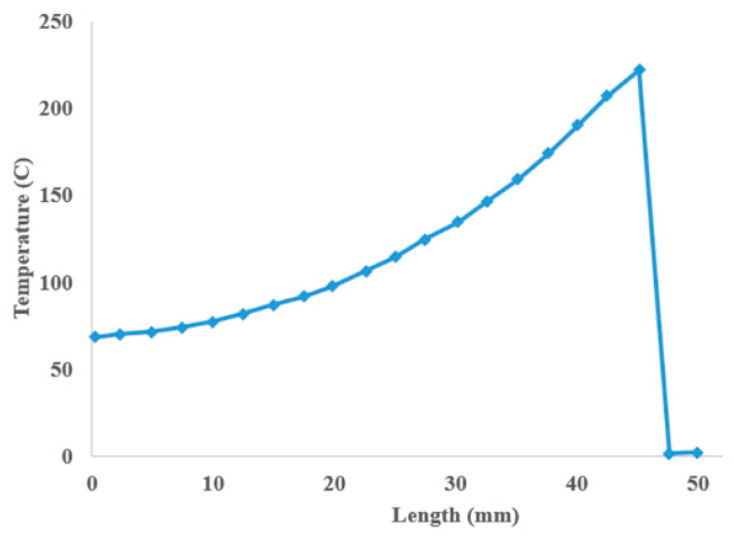
Temperature distribution along the filament length [77].

As the material properties of additively manufactured parts, either thermal or mechanical, are direction specific, the thermal conductivity is considered a tensor with three components of $k_{x}$, $k_{y}$, and $k_{z}$. During the phase transition of the material [78], considering latent heat in the analysis helps to simulate the heat transfer phenomena more accurately. The authors modeled the latent heat from phase transition as an internal change in enthalpy, as shown in Equation (30). The ANSYS SHELL281 element was used to mesh the platform and substrate, while the 20-node SOLID90 element was utilized to mesh the ABS filament. The authors considered one road of material filament in their analysis and made the following assumptions in their thermal analysis: (1) the temperature distribution is uniform throughout the filament cross-section, and (2) the filament length is considered semi-infinite. Figure 18 shows the nonlinear temperature distribution in an ABS filament during the FFF process, achieved from their FE analysis. Initially activated elements on the left side of the filament are about to reach a constant equilibrium temperature of about 60 °C, while the right side of the filament is not yet activated.

A 3D computational model was developed by Khanafer et al. [79] to accomplish transient heat transfer and investigate the inter-layer adhesion behavior. The authors then validated their proposed numerical model based on published experimental and analytical data. They utilized their numerical model to predict the temperature evolution in the interface region between layers. The temperature history was coupled with a mathematical model describing the bonding potential to predict the bonding formation initially proposed by Yardimci [80]. The authors developed the element activation process using the element birth-and-death technique available in the ANSYS software package. First, a very small stiffness is assigned to dead or inactive elements to play the role of molten material. Subsequently, after an element is activated during the analysis, the stiffness matrix is updated with the updated values of the element stiffness. The entire computational model containing all the nonlinearities, such as temperature-dependent physical and thermal properties, is controlled by preparing a code using the ANSYS APDL programming language. As previously discussed, the boundary conditions assigned to elements continuously change during the FFF process simulation, since newly activated elements influence the type of heat transfer occurring from the surface of previously deposited elements. These changes in the boundary conditions are controlled by subroutines written in the ANSYS programming design language (APDL).

Based on the results achieved from their numerical model, the researchers found that the chamber temperature had the greatest impact on the bonding potential, with the printing bed temperature being the second most effective parameter. Conduction heat transfer to the previously deposited adjacent material roads occurred at lower rates, mainly due to the rapid crystallization of polymers acting as a thermal insulation coating. They also showed that the material deposition in the newly added layer leads to a temperature increase in the adjacent layers, and this remelting of material at temperatures above the glass transition temperature is responsible for inter-layer bonding. The reheating slows during deposition, primarily due to the insulation behavior of the crystallized layers in between. Another parameter that affects the inter-layer melting and, subsequently, the degree of bonding is layer thickness.

Ravi et al. [81] proposed an in-process localized preheating technique to enhance the inter-layer bonding in the FFF process of polymers. They used a solid-state laser to preheat the substrate before new molten material was deposited. There are two heat transfer phenomena present in their study: the deposition and solidification of the molten material and the reheating of the previously deposited substrate using a laser. Therefore, the thermal profile and temperature distribution inside the material play crucial roles in the resulting micro-structure, mechanical properties, and final failure. The authors conducted a 3D transient thermal and heat transfer simulation-based model to better understand the thermal behavior of material being preheated by the laser. To construct their computational model, they used the FEM package COMSOL. Two principal models used for laser energy simulation in FEA are the Goldak and Gaussian energy profiles. The authors modeled the laser as a moving heat source over the substrate with a Gaussian energy input profile. To define the coupling between optical and thermal energy in the form of laser illumination on the substrate, they used two coefficients of absorption and reflectance. Considering a scanning laser beam across the solidified substrate made of black-colored ABS, they obtained the spatial temperature profile from a transient heat transfer simulation established in the commercial FE modeling software package COMSOL.

Among the thermal models utilizing the birth-and-death-of-elements function in ANSAS is the set of 3D simulations proposed by Ji and Zhou [10], which considers ABS as the material. They presented a three-dimensional nonlinear transient thermal finite element model for the FFF process using ANSYS APDL, considering latent heat enthalpy as the external heat source, as considered by many other researchers [44,55,77]. They showed that the temperature field distribution on the surface of the printed part resembles an ellipse, and the edges of the manufactured part experience the maximum temperature gradient.

Santos et al. [82] implemented a one-dimensional finite differential method using MATLAB to model the temperature evolution of an extruded filament. They then compared the results of their 1D model with two-dimensional COMSOL Multiphysics simulations. Finally, they experimentally validated the results of their simulation using infrared thermography. They considered ABS as the test material and used simplified thermal boundary conditions of forced convection and radiation heat losses to the surrounding atmosphere.

Zhou et al. [83] developed an experimental method to measure the temperature of PLA polymer during deposition using infrared sensors. Along with their experimental setup, they simulated an FE model in ANSYS 17.2 in order to predict temperature and stress distribution during the process. The authors investigated the effects of printing bed temperature, nozzle temperature, layer thickness, and printing speed on diffusion time and the maximum vertical distortion of printed parts. FE analysis results showed that the highest diffusion time occurred with a high nozzle temperature, high layer thickness, low printing speed, and high printing bed temperature. The experimental results confirmed these findings from their simulations. Furthermore, their verified model demonstrated that lowering the extrusion temperature, decreasing the layer thickness, and slowing the printing speed can reduce vertical distortion and thermally induced residual stresses.

Huo et al. [84] proposed a dual-temperature control method to improve the printability of polymers using poly(ε-caprolactone) as the test material. They considered the material’s viscosity, which varies with temperature and shear rate, to study the effects of two different temperature control modes. By utilizing dual-temperature control, they were able to reduce the width of the deposited poly(ε-caprolactone) filament to 50 μm. The experimental results validated their proposed model and suggested that dual-temperature control FFF can manufacture spatially arranged constructs and presents a promising application in the field of tissue engineering.

The meso-structure formed by parallel strands during the continuous non-isothermal deposition flow of PEEK and PLA polymers was comprehensively studied by Zhou et al. [85] using numerical simulations and experimental methods. They simulated the entire FFF process, covering polymer flow, material deposition, inter-layer bonding, and heat transfer for the materials under investigation. The results obtained from their model showed that increasing the reheating temperature can considerably improve the bonding between two strands. They extensively investigated and compared the effects of gap distance, printing speed, and strand-to-strand distance on the meso-structures of PEEK and PLA. Additionally, the simulation and experiment data provided detailed information regarding the porosity and bonding potential, highlighting their key roles in affecting the final product performance.

Costa et al. [86] studied overall heat transfer through various thermal phenomena involved in the FFF AM process, including convection and radiation with the surrounding atmosphere, conduction with support and between adjacent filaments, and radiation between adjacent filaments and convection with entrapped air, using Abaqus software. They adopted Newton’s method to solve nonlinear thermal equilibrium equations. For the heat transfer analysis, they utilized a total of 5240 elements of type DC3D8 with reduced integration points. They found out that heat dissipates primarily by convection with the environment and by conduction between adjacent filaments and the printing bed. Depending on the magnitude of the heat transfer coefficient, heat exchanges with the environment by radiation can be significant. However, the study found that radiation between adjacent filaments and convection with confined air can be neglected in practice. Also, the results showed that the temperature distribution across any filament cross-section is relatively uniform.

## 6. Structural Analysis

**Figure 19:**
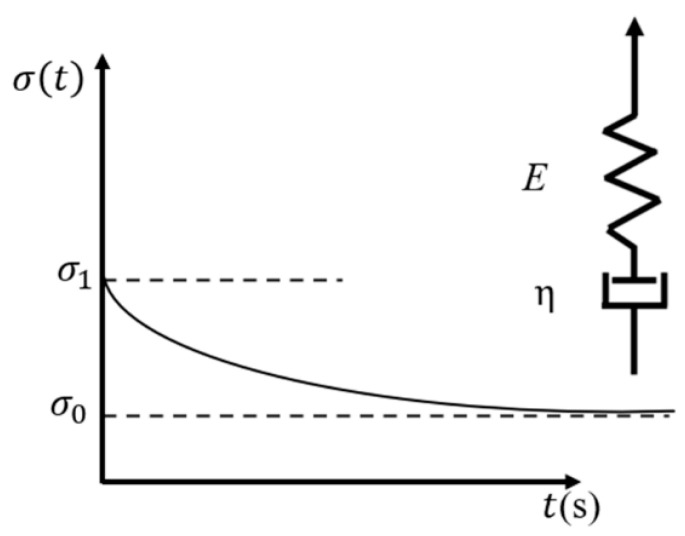
Schematic representation of the Maxwell model.

In the FFF process, a polymeric material flows from the nozzle in a semi-solid state and cools down to room temperature, solidifying into a fully solid part. In terms of mechanical material behavior, polymers occupy a middle ground between elastic solids like metals and fluids. They exhibit the solid characteristics of elastic materials and store elastic energy when experiencing external loading, while they dissipate a portion of the energy and flow on the micro-scale, showing a behavior like that of fluids [44]. Several physical models have been developed by researchers to describe the linear viscoelastic behavior of polymers. For example, the Rouse model uses the Brownian motion theory to simulate the single-chain diffusion of beads that are connected via harmonic springs. The Kremer–Grest model uses several hundred chains to simulate polymer elements. Other single-chain theories such as the tube theory and the arm retraction model with arm starts have also been developed to describe the linear viscoelastic behavior of entangled polymers. Among these models, the generalized Maxwell model is widely recognized as the most frequently utilized model for describing the glass transition of linear viscoelastic solids [87]. The single-element Maxwell model uses a spring and dashpot connected in series to represent the elastic and viscous behavior of the material, as shown in Figure 19 and defined as in Equation (32) [44]:

(32) $$\sigma (t)=\varepsilon_{0}\cdot E(t)=\varepsilon_{0}\cdot E\cdot \exp\left(-\frac{t}{\lambda }\right)$$

where λ is the relaxation time, and *E*(*t*) is the relaxation modulus. Considering only one element in the Maxwell model is not sufficient to describe realistic material behavior. Hence, multiple Maxwell models are usually considered in parallel, which is known as the generalized Maxwell model and is defined as

(33) $$\sigma (t)=\varepsilon_{0}\left(\sum_{i=1}^{n}E_{i}\exp\left(-\frac{t}{\lambda_{i}}\right)+E_{\infty }\right)$$

where *n* is the number of Maxwell models considered in parallel, which indicates the model’s accuracy in analysis. Brenken [44] extended the generalized Maxwell model by considering 29 parallel elements (*i* = 29 in Equation (33)) to simulate the viscoelastic behavior of carbon fiber-reinforced polyphenylene sulfide (PPS) manufactured by FFF additive manufacturing. He implemented the material behavior in a series of user subroutines using Abaqus software and investigated the distribution of residual stresses and distortion by considering a carbon fiber-reinforced PPS plate in his simulations and experiments. In order to examine his FE simulations and proposed numerical methodology, he printed an air inlet duct, setting the print bed temperature to 180 °C and the extrusion temperature to 300 °C for a PPS composite. To simulate the print bed temperature, he applied a constant temperature of 180 °C to the bottom nodes of the model in direct contact with the bed. These nodes were also fixed in three directions to simulate the print bed’s mechanical boundary conditions. To verify the accuracy of his FE model, he considered a simple plate with dimensions 120 × 120 mm^2^, comprising a total of four layers; the first two layers were printed at 0° and the last two layers at 90°. He compared the deformation magnitude in the printed plate from FE simulations with experimental measurements and found the proposed model to be a useful tool for predicting warpage in additively manufactured components.

Favaloro et al. [88] utilized recently added features to newer versions of Abaqus (2017 and after), such as element activation and event series, to simulate polymeric composite additive manufacturing. They explain in detail the sequentially coupled thermo-mechanical analysis of the FFF process developed by Brenken [44]. Progressive element activation, which is key to extrusion-based physical systems such as FFF (sometimes referred to as fused filament fabrication, FFF) is performed by employing the user subroutine UEPActivationVol. They used the same material as in Ref. [44], a semi-crystalline composite polymer that can achieve a maximum relative crystallinity of 84%. Fiber-reinforced semi-crystalline composite polymers have different material properties in different directions, and hence, material direction needs to be considered while simulating these types of materials. The authors modeled the thermoplastic behavior of CF PPS using a user subroutine structure that performs element activation based on machine instructions and provided tool path from G-Code. This structure assigns appropriate local coordinate systems to account for aforementioned anisotropic material properties.

First, the ORIENT user subroutine is called at the beginning of the analysis to assign material direction to each element based on the nodal coordinates and the information provided in the tool path. Another functionality of ORIENT is to assign the activation time of each element by extracting timing information from the tool path provided. The material direction and element activation time calculated in ORIENT are stored in the UEXTERNALDB user subroutine for the first integration point of each element. These data are then shared with other subroutines that need this information through analysis. These data are then used in the following user subroutines: ORIENT, in which to avoid extensive calculations for each integration point, the saved data for the first integration point of each element in UEXTERNALDB is assigned to the other seven integration points (in the case of using eight-point elements) of that element in this user subroutine; DSVINI, which is usually used to initialize state variables in Abaqus and needs access to the activation time of each element; and UEPActivationVol, a user subroutine that, as stated above, is employed to activate the elements in a certain order based on their activation time, which is provided by UEXTERNALDB.

After the completion of element activation, the thermal analysis is implemented in the UMATHT subroutine. The degree of crystallinity is temperature dependent and therefore is defined in UMATHT after temperature calculation. Alternatively, crystallization can be defined inside the UEXPAN user subroutine as well. Because thermal strain depends on the degree of crystallinity, and these strains need to be defined inside UEXPAN, it is beneficial to define the degree of crystallinity inside UEXPAN. The authors used UMAT to implement the mechanical analysis and define the material’s mechanical properties. They presented the dependence of material properties on the degree of crystallinity, the anisotropic nature of such materials, and the shrinkage and shape changes during crystallization.

Samy et al. [89] discussed the effect of various printing parameters, such as build platform temperature, layer adhesion, layer thickness, and printing pattern, on built-up residual stresses and warpages using COMSOL. They correlated crystallization kinetics and viscoelastic and thermo-mechanical properties with temperature changes during FFF using element activation in COMSOL. During the FFF process, the materials solidify at different rates depending on the surrounding temperature, which makes element activation a suitable technique for simulations. The authors’ COMSOL simulations demonstrated that a decrease in layer thickness reduces warpage and residual stresses. A line raster pattern can reduce warpage and residual stresses by 16% and 36%, respectively. They used the generalized Maxwell model to mimic the thermo-viscoelastic behavior of the polymer. Their multiphysics simulation of FFF in COMSOL included solid mechanics, heat transfer analysis, and polymer crystallization kinetics. They defined all the thermo-mechanical properties of the material, such as specific heat capacity, thermal conductivity, and density, as a function of temperature. Then, the temperature profile was used to calculate the degree of crystallinity, affecting the residual stresses and consequently the resulting warpage and deformation. Mesh optimization is crucial, and the element size must not exceed the nozzle diameter, and the height dimension must not exceed the layer height to achieve accurate and reliable results. In their work, the authors used 0.5 × 0.5 × 0.1 mm³ elements to match a 0.1 mm layer height and 0.5 mm bead width. Boundary conditions included a print bed temperature of 100 °C, extrusion temperature of 210 °C, and a spring foundation instead of fully fixed BC to allow for warping. This approach provided a more accurate simulation of the actual printing process.

**Figure 20:**
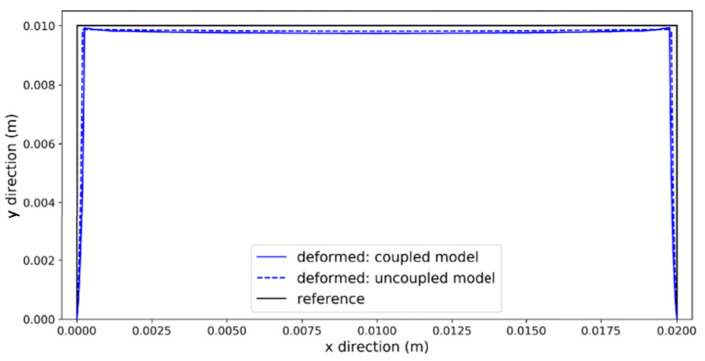
Reference and final configurations of the two-dimensional wall for the coupled and uncoupled models. Reprinted from [90].

Wang and Papadopoulos [90] proposed a novel FE simulation method for fused deposition modeling additive manufacturing in two dimensions. Unlike many other studies that consider sequentially coupled thermo-mechanical analysis, the authors assumed full coupling between the mechanical and thermal responses by simplifying the problem with two assumptions: infinitesimal deformation and finite temperature variation. Material shrinkage in the FFF process from the molten state to the solidified form is not negligible [44], justifying the fully coupled thermo-mechanical analysis considered by [90]. In a fully coupled thermo-mechanical analysis, not only does the thermal analysis affect the mechanical analysis, but the mechanical analysis also affects the thermal simulation. For efficiency, the authors implemented their FE method in a stand-alone C++ code using a highly efficient package called UMFPACK (version 5.1) to solve the unsymmetric linear-algebraic system of equations. To validate their FE model’s accuracy and reliability, they conducted a preliminary analysis of a simple two-dimensional printing problem. The printed part was a rectangular plate measuring 20 mm in width and 10 mm in height with 40 elements along the x-direction and 30 elements in the y-direction. The applied boundary conditions considered were: Dirichlet boundary conditions with zero nodal displacement in both the *x*- and *y*- directions to the bottom side, a constant ambient temperature of 315 K, no traction on the other three sides of the plate that were subjected to convection heat transfer conditions exposed to the ambient temperature, and a material extrusion temperature of 500 K. Finally, they compared the temperature and displacement distributions from their fully coupled thermo-mechanical model with those from uncoupled analysis, as shown in Figure 20.

Moumen et al. [91] investigated residual stress, stress concentration, distortion, and delamination between layers in tensile test specimens made of a polyamide (PA 12) polymer composite. They developed a 3D thermo-mechanical model to simulate the FFF process, capable of calculating stresses and temperature gradients during additive manufacturing. The model considers temperature-dependent physical properties such as density, thermal conductivity, thermal expansion coefficient, yield stress, and Young’s modulus. The simulation includes the heating, solidification, and cooling phases. The authors used the element activation/deactivation capability of Digimat 2018.1 software to simulate the FFF process for composite parts. They employed a coupled thermo-mechanical element type for both thermal and mechanical analyses. Temperature distribution obtained from thermal analysis was used to calculate residual stresses in the printed part. Material deposition followed two methods: bead-wise and layer-wise, guided by the tool path extracted form Slic3r software’s G-Code files. These files include time and spatial position of the extruder head, polymer deposition description, and the part’s base and contour. As opposed to studies focusing only on bead-wise activation (line-wise material activation), the authors considered both bead-wise and layer-wise material deposition methods. They compared their numerical analysis results with experimental data, showing a maximum discrepancy of 5%, which shows the effectiveness of numerical simulations in the thermal and structural analysis of the FFF process.

Xia et al. [92] developed a multi-scale algorithm to describe the FFF additive manufacturing process, incorporating the conductive heat transfer and solidification models. Their proposed system simulates material states at both the macro-scale and micro-scale, capturing multiple physical phenomena. In the mesoscopic case, the authors focused mainly on changes in thermal conductivities caused by temperature-induced warpage and considered the anisotropy feature of crystal formation and growth at the micro-scale. The model can capture multiple physical phenomena such as the cooling, solidification, crystallization, and deposition of the filament. The novelty of their work is a fully integrated simulation platform that considers different spatial scales to build a digital model of the real manufacturing process. They first introduced multi-scale governing equations such as a Lyapunov-type energy function to describe the coupling between the phase field and conductive temperature field. The numerical solution of the multi-scale system was discretized using a uniform mesh grid in two dimensions, which can be extended to three dimensions. They used the Fourier spectrum method to solve these equations. For the macro-scale formulation, they used the pressure correction method, while for the micro-scale formulations, they performed the invariant energy quadratization (IEQ) method to transform their original micro-scale system.

**Figure 21:**
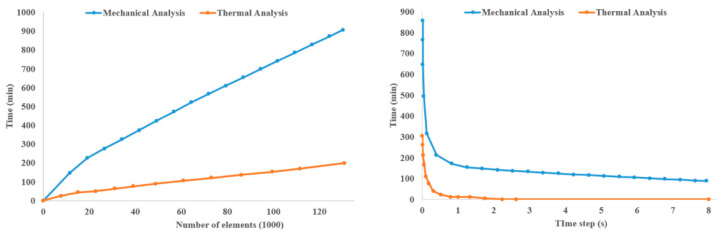
Mesh size effect (**left**) and time step effect (**right**) on different types of analysis [93].

Cattenone et al. [93] conducted a comprehensive numerical study on mesoscopic and macroscopic simulations of the FFF process using the Abaqus software package. After describing the methodology, they investigated the impact of various print and process parameters and modeling configurations on the simulation results and validated these results with experimental measurements. They prepared a G-Code file for the part of interest, which was then used both to print the part physically and to implement the print simulation in Abaqus, where the tool path was required for element activation. They first completed the heat transfer analysis and then the coupled thermal analysis with structural analysis sequentially to capture deformation and warpage resulting from temperature gradients. They tested their numerical model with different element sizes and time steps and found a linear relationship between total mechanical and thermal analysis time and the total number of elements, as shown in the figures below. However, the relationship between total analysis time and time step was not necessarily linear, as shown in Figure 21.

Considering different meshing strategies, they reached the following conclusions: (1) A finer meshing scheme fully consistent with the filament dimensions (printer physical configuration) allows for a more accurate prediction of stress gradients, even though it significantly increases computational cost. (2) For small models, where local effects can significantly affect part deformation and warpage, users are encouraged to utilize finer element sizes. (3) For large models, where the local effects are negligible compared to the global dimensions of the part, a coarser mesh size is suggested to reduce computational costs. Finally, they compared the stress distribution, deformation, and temperature profiles obtained from their simulation with experimental data by printing two different geometries, a planar spring and a bridge model, to validate the accuracy of proposed model.

Zhang and Chou [94] investigated the FFF process, which encompasses complicated heat and mass transfer phenomena fully coupled with mechanical loading and phase changes. They developed a finite element analysis-based model using the element activation function in ANSYS software to simulate the mechanical and thermal phenomena in FFF and later utilized it to simulate temperature-induced residual stresses and part distortion. They used solid structural elements for coupled thermo-mechanical analysis, as there is no element type in ANSYS that can simultaneously meet the requirements of thermal and mechanical analyses and support the viscoelastic features of semi-solid material. One simplifying assumption they made is that the newly deposited material was assumed to be fully in contact with the substrate (previously added material or print bed), neglecting the small space between the extruded beads of material. They also used their proposed model to study the tool-path effects on the FFF process. After printing several simple plates with different raster patterns, they observed that the tool-path pattern affects both the magnitude and distribution of temperature-induced residual stresses, with stress concentrations primarily aligned in the direction of the tool path.

In another study, Zhang and Chou [95] conducted a series of FEA simulations to investigate the effect of different print parameters on part distortion during the FFF process. They utilized the commercial software ANSYS to develop sequentially coupled thermo-mechanical analysis and prepare simulation codes. The elements selected were rectangular parallelepipeds with dual attributes (solid 45/solid 70), compatible with both thermal and mechanical analyses. Their thermal analysis was governed by the transient heat conduction equation incorporating heat generated from phase changes in three dimensions. The bottom surface of the model, in direct contact with the printing bed, maintained a constant chamber temperature of 75 °C. Forced convection was assigned as a boundary condition on the other outer surfaces. From their simulation results, the authors found that scan speed and layer thickness had the most significant effects on part distortions. Additionally, they observed that the interaction between the road width and the layer thickness can significantly influence part distortion. In general, printing with thick and wide roads results in greater residual stresses in the FFF process.

Yang and Zhang [96] established a numerical model to analyze temperature and stress distribution during the forming process of fused deposition modeling using the finite element method and the element birth–death technique. They first solved the thermal analysis and used the nodal temperature as a predefined field for the structural analysis. The SOLID70 element type was selected for performing 3D steady-state or transient thermal analysis, while the SOLID185 element type was chosen for structure analysis. Since the 3D printing process of polymeric materials like PLA transitions through different states from molten to semi-solid and solid states, this element type is suitable for analyzing plastic, superelastic, stress-stiffening, and large deformation processes. They assessed various infill patterns and concluded that the honeycomb infill pattern is optimal because it results in minimal temperature gradients, uniform stress distribution, and reduced deformation in printed parts.

Talagani et al. [97] conducted a simulation of the additive manufacturing process of a full-sized car using Abaqus and GENOA software. GENOA was used to automatically create a mesh of moderate size based on the tool path from the G-Code file, generating a regular rectangular mesh. The entire car model was composed of 419,759 elements. Coupled thermal-structural analysis in additive manufacturing can be implemented in three general forms: (1) Sequentially coupled thermo-mechanical analysis is used when only the structural analysis depends on the temperature field and thermal analysis is independent of the structural analysis. (2) Fully coupled thermo-mechanical analysis is performed using the temperature field obtained from experiments. (3) Fully coupled thermo-mechanical analysis is performed when there is a strong mutual interaction of the mechanical and thermal solutions. This requires the use of elements with both displacement and temperature degrees of freedom in the model. The authors used the fully coupled thermal–mechanical analysis, as they had access to the temperature distribution from the tests. Using the capabilities of GENOA, they also implemented failure analysis. Finally, they obtained the temperature distribution, residual stresses, potential crack initiation, and crack growth sites from their analysis.

As the second part of their comprehensive study on FFF process simulation, Xia et al. [34] proposed a numerical model to predict residual stresses and deformation introduced to the FFF parts due to temperature gradients inherent to the FFF process. The behavior of a polymer melt can be modeled using mass conservation, momentum conservation, and energy equations, as presented previously in Section 2. To model material shrinkage and volume changes, the density of the polymer is considered a function of temperature and pressure. However, as stated in their previous study, since the material reaches atmospheric pressure after flowing out of the nozzle, the pressure remains constant, and the only factor that affects the density is temperature. The nozzle is defined as a cylinder with one end closed and the other end open. The boundary conditions are the same as those described previously. The proposed model is able to predict stress, material shrinkage, and deformation for small parts.

Xu et al. [98] presented a non-isothermal viscoelastic computational fluid dynamics model to determine the effect of steady and transient feeding forces, the phase transition process, and the viscoelastic behavior of semi-crystalline polymers on the FFF process. They conducted all numerical simulations of flow behavior, heat transfer, and structural analysis using ANSYS Fluent. The finite volume method was employed to solve the CFD model within the software. A user design function was developed in the C++ programming language to implement the viscoelastic constitutive model. The results obtained from the proposed model demonstrate that the effect of elasticity on semi-solid material being extruded is more influential compared to the effect of viscosity, especially at high extrusion rates, and results in greater residual stresses in the extruded filaments of material.

## 7. Material Characterization

Abeykoon et al. [40] focused on studying the mechanical, thermal, and morphological properties of 3D-printed parts with varying processing parameters such as infill pattern, infill density, and infill speed using different printing materials, including polylactic acid (PLA), acrylonitrile butadiene styrene (ABS), carbon fiber-reinforced PLA (CFR-PLA), carbon fiber-reinforced ABS (CFR-ABS), and carbon nanotube ABS (CNT-ABS). The authors implemented a series of FE analyses to visualize the stress profile in simple tensile, bending, and compression tests of the PLA test specimens. The 3D solid models of the samples were created using the SolidWorks software and fully analyzed in the ANSYS multiphysics software, a robust and reliable package for thermal and structural analyses. Applying the correct boundary conditions is crucial in FE analysis, as these directly influence the results. To replicate the same testing and boundary conditions, the authors set up the samples in the exact same way that the samples were held in the tensile and compression testing machine and the three-point bending setup. Tetrahedral elements were used for the meshing of solid models in their study.

**Figure 22:**
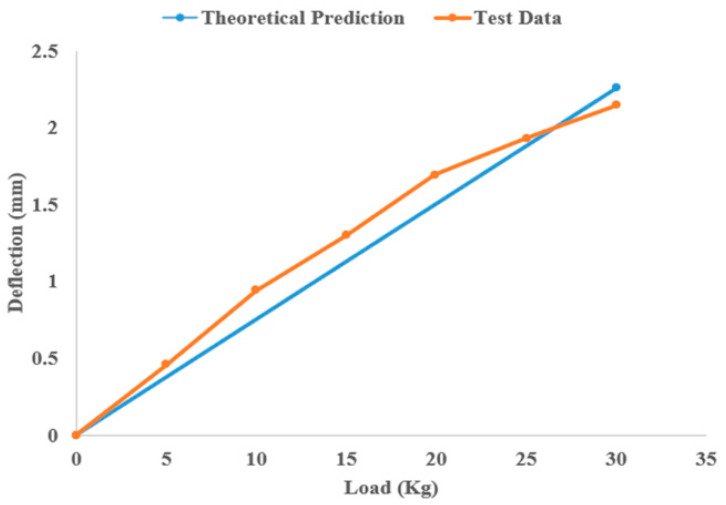
Testing of the small-size UAV frame: experiments versus theoretical results [99].

Azarov et al. [99] presented a complete product development cycle for a small-size unmanned aerial vehicle (UAV) frame made of carbon fiber-reinforced composite (CFRC) material via FFF additive manufacturing. The development cycle includes design, mechanical property determination, FE simulation, and mechanical testing of the frame. They first extracted the properties of PLA material containing 20 percent carbon fiber by conducting tensile and three-point bending experiments. Then, they used the obtained properties to conduct simulations and stress analysis in the Siemens NX package and NX Nastran CAE module. The stiffness of the material in the fiber direction and the Poisson ratio were obtained from the tensile test, while the interlaminar shear modulus was derived from the three-point bending test using Equation (34):

(34) $$w_{\max}=\frac{PL}{4}\left(\frac{1}{S}+\frac{L^{2}}{12D}\right)$$

where *S* = *5 Gbh*/6, *D* = 5 *Ebh*^3^/12 for a laminated beam of rectangular cross-section (h × b), *P* is the applied load, and *w_max_* is the maximum deflection. The rest of the mechanical properties of the composite material were approximated using the aforementioned properties and the properties of pure PLA as the matrix material. Due to the thin structure of the UAV, shell elements were used in finite element simulations, and the load applied was applied in the form of uniform pressure in the middle section of the frame. The maximum applied load and the maximum deformation experienced by the frame were compared to the results from experiments. The results show the accuracy and liability of the FE simulations, as shown in Figure 22.

In another study, Somireddy and Czekanski [100] applied a computational homogenization technique to estimate the constitutive behavior of additive manufactured polymeric composite parts considering carbon fiber-reinforced ABS (CF ABS) as the material used. Due to the layered structure inherent in their manufacturing process, FFF parts can be considered composites. The material behavior of layers of these 3D printed structures can be considered orthotropic, allowing for the application of the stress–strain relationship for an orthotropic material. The authors considered their FE model of the representative volume element (RVE) for 3D-printed components made of CF ABS to perform computational homogenization. The simulation results were then used to calculate the effective stiffness matrix and elastic moduli. The results indicate that the RVE showed maximum stiffness in the print direction, where all fibers were aligned.

**Figure 23:**
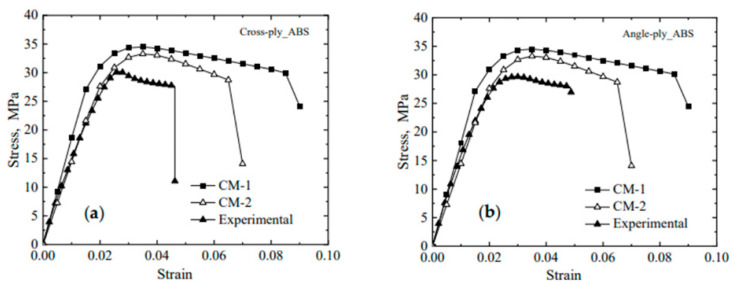
Stress–strain curves of 3D-printed parts: (**a**) cross-ply and (**b**) angle-ply patterns. Reprinted from [101].

Somireddy et al. [101] proposed two different computational methodologies for modeling the failure behavior of 3D-printed parts,. They considered parts made of two materials: pure ABS and CF-ABS containing 20 percent carbon fibers under uniaxial loading. In the first method, they used the nonlinear properties of the virgin material in their computational model, which predicted higher values than the experimental results. This method employed an isotropic damage and plasticity law. The FE models of the representative volume element (RVE) assumed perfect bonding at the interfaces of adjacent beads. Other simplifying assumptions included perfect bonding between fibers and the matrix, the perfect alignment of fibers in the print direction, and the sparse distribution of carbon fibers throughout the matrix (pure ABS). These assumptions idealized the computational model. The significant difference between experimental and computational results becomes evident especially in the case of 3D-printed composites because the properties of printed materials cannot be accurately estimated with the properties of traditionally manufactured components (virgin material). In the second method, however, the computational model utilized nonlinear material properties obtained from the mechanical testing results of sample printed parts, accurately predicting the nonlinear behavior of 3D-printed parts. The authors therefore suggest using this methodology for effective design and analysis to capture accurate failure behavior. Figure 23 compares the stress–strain behavior of materials obtained from both computational models with experimental results, clearly showing that the second model predicts the material behavior more accurately.

Mishra et al. [102] studied the effects of layer thickness on the impact, flexural, and tensile strength of 3D-printed polyamide specimens. They developed a finite element model to predict the tensile and flexural strengths of printed specimens with over 87 percent accuracy compared to experimental results. The authors used ANSYS 2022 R2 for their simulations, with elastoplastic deformation assumptions for bending and tensile specimens under three-point bending and uniaxial tensile loads, respectively. Implicit analysis was chosen for the elastoplastic computations, as it typically produces better results compared to explicit analysis despite its higher processing time and memory. Tetrahedral elements were used to mesh the model, with a comparatively fine mesh chosen based on the layer thickness. Since the height of the bead of material activated at a time equals the layer thickness, the mesh size in the stacking direction should not exceed the layer height. A mesh size of 1 mm was selected based on simulations with various sizes. Boundary conditions were then applied to replicate the real testing mechanisms. The authors evaluated the effect of layer height on tensile and bending stress and the effect of infill density on the impact strength and achieved results comparable to experimental data.

Employing FE analysis, Seibert et al. [103] proposed a robust model to validate the averaged strain energy density (ASED) criterion applied in the failure prediction of cracked PLA parts manufactured by FFF additive manufacturing. The authors obtained experimental data, and the results were compared to the theory of critical distances (TCD) to further evaluate the accuracy of their model. To determine the strain energy density field, they conducted tensile and bending tests on the notched specimens via finite element simulation using ABAQUS for meshing the model and solving the constitutive material equations. They employed quadratic elements and a linear elastic isotropic material model with the material properties obtained from experiments. As previously mentioned, the time and CPU efficiency were obtained using symmetry in the finite element simulation. Therefore, the authors employed quarter models and half models to exploit the symmetry in the tensile and bending tests, respectively. Although large mesh sizes are also suitable for the ASED criterion, they used a very fine mesh with seed distances proportional to the notch root radius. The authors selected 2D elements for the computations and conducted both plane stress and plane strain analyses to demonstrate the robustness of the presented methods. Through their experiments, the authors found that the plane stress assumption is more accurate for *ρ* > 1 mm, whereas the plane strain assumption gives more accurate results for *ρ* < 1 mm, with *ρ* being the notch root radius. Since small notches result in a higher stress concentration and are more prone to failure compared to large cracks, the plane strain assumption was used throughout these analyses to account for both large and small discontinuities.

Domingo-Espin et al. [104] conducted a simulation-based study to develop a numerical model for FFF parts made of polycarbonate (PC) under bending and torsion loading and correlated the FEA simulation with experiments. Treating FFF parts as orthotropic materials, they determined the nine mechanical constants that define the stiffness matrix of an orthotropic material through the experimental tensile testing of specimens. Young’s moduli, Poisson’s ratios, and shear moduli were experimentally obtained along with the yield strength and ultimate tensile strength of each specimen for later use in simulation. They correlated the simulation results with physical testing (tensile, bending, and torsion) by designing and manufacturing simple parts in different orientations. They then used the stiffness matrix for the orthotropic constitutive model to simulate the mechanical response of the physically tested parts. The data for the stiffness matrix were obtained from the mechanical characterization. They conducted seven different simulations for each specific orientation tested physically and one simulation assuming that isotropic material properties were the same in all direction. The mechanical constants in the isotropic simulation were obtained using the mean values of elastic modulus, Poisson’s ratio, and shear modulus in the print direction, in-plane shear direction, and build direction.

ANSYS Mechanical 15.0 software was used to simulate the deformation of a part considering the building orientation. The boundary conditions for the FEA simulation were established according to the physical test conditions [40]. A realistic boundary condition applied by the authors was the contact defined in the area of load application in bending, as shown in the figure above. This is because the applied load will not remain vertical due to the bending of the arm. Such boundary conditions help align the simulation and experimental results. Based on how the parts were built, the mechanical constants were set in each simulation. In their FE model, the authors assumed that the material behavior remained in a linear regime (elastic region in the stress–strain curve) and the loading was static. A hexahedral mesh of a Solid 185 element type with a total element number of 10,476 and a total node number of 14,008 was used for all loading cases in the model. One of the outstanding findings of their simulation model is that anisotropic material properties should be used in the FEA simulations of FFF parts when plastic deformation occurs and loading goes beyond the elastic region. On the other hand, while in the elastic regime, the material can be considered isotropic using the average values of the mechanical properties. This is an important result since an isotropic model is easier and more convenient to implement. Furthermore, there is no need to modify constitutive equations in the materials database of FEA software or to deal with material orientations in the model.

Scapin and Peroni [105] developed a numerical tool for designers to predict the actual mechanical properties of FFF-printed parts through finite element analyses. They considered different composite polymers such as glass- and carbon fiber-reinforced nylon as testing materials. By performing tensile tests on 100% filled dog bone specimens in two different directions, they obtained the elastic parameters of the printed material. They then used these parameters in numerical analyses to predict the mechanical responses of the test specimens. Numerical simulations were performed using an LS-DYNA code employing nonlinear static implicit analyses. Shell elements with fully integrated formulations were adopted in their analysis due to the low thickness of the printed specimens. The results of their proposed model demonstrated that the purely elastic transversely isotropic material model can accurately predict the behavior of 3D-printed parts before nonlinearities occur.

Bouaziz et al. [106] developed a combined computational and experimental methodology to study the fracture behavior of 3D-printed ABS specimens using the FFF AM technique. They used digital image correlation (DIC) combined with FE simulations in Abaqus to evaluate the J-integral as a fracture mechanics parameter by analyzing displacement and strain field around the crack tip. Micro-scale empirical analysis performed via DIC helped to measure surface displacement and strain fields. In their study, the authors used two independent methods to assess the crack tip location and J-integral. First, FE calculations were performed in ABAQUS using measured displacements from DIC experiments as boundary conditions. The crack tip position was found by minimizing the error between computed and measured displacement fields. Then, the authors implemented MATLAB scripts to find the crack tip position and calculated the J-integral using the measured kinematic fields.

Bonada et al. [107] analyzed the influence of different infill patterns on the mechanical properties of FFF-printed parts. The elastic constants of the material (Young’s modulus and Poisson’s ratio) were obtained from experiments and used for the numerical analysis and simulations. The authors performed FE analyses using ANSYS 2021R1 software using the Solid 185 element type to mesh the representative volume element (RVE) domain. To avoid mesh dependency in the FEA results, they used refined meshes with a minimum number of 62,000 elements in each RVE model. The results showed that the grid infill pattern resulted in the weakest mechanical properties. Therefore, using 3D-printed components with such an infill pattern is not recommended for applications requiring high stiffness and optimum tensile strength. Moreover, FFF-printed components with a [0 90°] raster pattern have larger Young’s moduli compared to those printed with a linear 45° infill pattern.

The numerical approach developed by Dialami et al. [108] has the capability to predict the elastic properties of FFF-printed components with different printing (infill) patterns. They used the open-source Kratos Multiphysics finite element code to perform computational homogenization analysis. This methodology significantly reduces the number of experimental tests necessary for characterizing the 3D-printed parts. After performing the finite element simulations for material homogenization, the nine unknowns of the orthotropic matrix could be obtained.

Yao et al. [109] experimentally and numerically studied the effects of micro-structure on the elastic properties of 3D printing materials and the vibration characteristics of simple printed plates via FFF. In their study, they used the FE software ABAQUS to carry out finite element simulations of 3D-printed plates made of PLA-max. Because of the small thickness-to-width ratio of the test plates, the authors selected the linear shell element (S4R) to implement the simulation models of the plates. In another set of simulations, they established their model based on the linear 3D stress element C3D8R to verify the accuracy of their original model using shell elements. Three different mesh sizes of 0.5 mm, 1 mm, and 5 mm were adopted to ensure calculation accuracy, verify convergence, and eliminate the mesh size dependency of the model. Simulations demonstrated that the largest difference in natural frequencies among plates with different printing directions when the mode of the plates changed from one to five were 10.645 Hz, 39.588 Hz, 66.710 Hz, 123.780 Hz, and 185.720 Hz, respectively. Moreover, these differences for in-plane natural frequencies are relatively large compared to those for the first five out-of-plane natural frequencies of these plates.

Birosz et al. [110] implemented a set of simulations to investigate the creep properties of a compressor wheel made of PLA manufactured by the FFF process. In order to reduce the complexity of extracting deformation data from experiments, they used ANSYS 2019 R3 FEM software to calculate the creep strains and deformations resulting from applied loads. Since linear models are unable to capture residual strain and large deformations at elevated temperatures, multilinear isotropic hardening and modified time hardening models were used to create the finite element model in ANSYS. The results of their simulations showed that the strain of the additively manufactured compressor wheel is not significant.

## 8. Conclusions

After reviewing and analyzing over 100 research articles contributing to the finite element (FE) analysis and numerical modeling of fused deposition fabrication (FFF) additive manufacturing (AM), we provide a comprehensive summary of the available and utilized FE software packages, numerical models, programming languages, and other useful tools in these research articles in tabular form, as presented in Table 1. From a numerical modeling perspective, this review paper thoroughly discusses and analyses six key aspects of the FFF process: melt flow, heat transfer and thermal analysis, solidification and crystallization, bonding efficiency, structural analysis, and material characterization.

There have been numerous studies on the melt flow behavior, thermal analysis, and structural analysis of the FFF process. However, as thoroughly studied and discussed in this review and evident from the summary table (Table 1), two very crucial aspects of the FFF process that influence the integrity and functionality of printed parts—solidification and crystallization along with bonding efficiency—still lack reliable numerical models and need further attention from researchers.

Temperature plays the most crucial role during the FFF process, as temperature drops cause the material to shrink and solidify. As the material shrinks, residual stresses begin to build up and could lead to part failures. Therefore, special attention needs to be paid and more effective models need to be developed to better understand the solidification phenomenon in the FFF process.

Additionally, all AM processes, including FFF, build components layer by layer. If these layers are not bonded together effectively, it leads to printed components with low integrity and reduced functionality. To address this issue, research should focus on the inter-layer bonding efficiency, and more studies are needed to further investigate inter-layer bonding efficiency during the FFF process.

The proposed models for thermal and bonding efficiency in FFF processes, while advancing our understanding, show several drawbacks and gaps that suggest directions for future research. Oversimplified methodologies, such as Jensen et al.’s 2D approach or Zhou et al.’s 3D analyses, often assume idealized conditions like uniform temperature distribution or constant material properties, which may not accurately capture the complex thermal behaviors and phase transitions observed in real-world scenarios. Models that focus solely on conduction or specific heating techniques, such as Ravi et al.’s localized preheating or Huo et al.’s dual-temperature control, may overlook the combined effects of convective and radiative heat transfer, as noted by Costa et al. and others. Additionally, several numerical models, such as those implemented by Ji and Zhou and by Santos et al., rely on assumptions that may oversimplify the interactions between material properties and thermal dynamics, potentially leading to inaccuracies in predicting the bonding efficiency and structural integrity of printed parts. Future research should address these limitations by developing more comprehensive and adaptive models that integrate multi-dimensional thermal phenomena, real-time process variations, and material-specific behaviors. Emphasizing experimental validation and exploring advanced simulation techniques capable of incorporating real-time monitoring and adaptive control strategies will be essential for bridging these gaps and improving the accuracy and functionality of FFF thermal and bonding effectiveness numerical models.

In conclusion, this review paper comprehensively presents a wide variety of FE simulation and numerical models applied at various stages involved in the FFF process. By studying and deeply analyzing over 100 references, we highlight the critical advancements and methodologies that enhance the overall efficacy of FFF additive manufacturing. These insights offer a valuable resource for scholarly researchers in academia and industry to develop novel models aimed at enhancing the FFF process. The detailed analysis presented in this study provides a robust foundation for future research, encouraging further enhancement and innovation in the field of additive manufacturing.

## Figures and Tables

**Table 1:** Summary of the numerical models and FE packages frequently used in the state of the art of the FFF process.

Mechanism	Numerical Models	FE Packages	References
Melt Flow Behavior	Navier–Stokes Power law Carreau–Yasuda Voigt model Maxwell model Kelvin model Cross-WLF	Ansys FLOTRAN Ansys CFD Ansys CFX Ansys FLUENT	[21,22,23,24,25,26,27,28,29,30,31,32,33,34,35,36,37,38,39,40]
Solidification and Crystallization	Avrami Ozawa Nakamura	COMSOL MATLAB ABAQUS	[41,42,43,44,45,46,47,48,49,50,51,52,53,54]
Thermal Analysis	Fourier’s law First law of thermodynamics	ANSYS ABAQUS MATLAB COMSOL	[55,56,57,58,59,60,61,62,63,64,65,66,67,68,69,70,71]
Bonding Efficiency	Yang Arrhenius	ABAQUS MINITAB	[72,73,74,75,76,77,78,79,80,81,82,83,84]
Structural Analysis	Rouse Maxwell Kremer–Grest	ABAQUS MATLAB COMSOL UMFPACK C++ DIGIMAT ANSYS GENOA	[85,86,87,88,89,90,91,92,93,94,95,96,97,98]
Material Characterization		NASTRAN ANSYS ABAQUS LS-DYNA MATLAB	[99,100,101,102,103,104,105,106,107,108,109,110]

## Data Availability

The original contributions presented in the study are included in the article, further inquiries can be directed to the corresponding author.
